# A method for determining the optimum supporting opportunity of roadway based on energy dissipation

**DOI:** 10.1371/journal.pone.0295533

**Published:** 2023-12-07

**Authors:** Qinzheng Wu, Chao Peng, Yang Liu, Danli Li, Haoqin Zhang

**Affiliations:** 1 Deep Mining Laboratory of Shandong Gold Group Co., Ltd. Laizhou, Yantai, China; 2 School of Resource Environment and Safety Engineering, University of South China, Hengyang, China; University of Science and Technology Beijing, CHINA

## Abstract

The time and space of the support structure applying is related to the overall stability of the roadway after excavation directly. Designed twenty-four groups of roadway support schemes with time and space dimensions, and studied the stability characteristics of roadway in different schemes by using Fast Lagrangian Analysis of Continua in Three Dimensions. The main conclusions are as follows: the influence of energy dissipation (time) and support position (space) on the stability of the roadway is not a linear relationship, and supporting at the appropriate opportunity can be beneficial. Established the "displacement-dissipation energy" curve, founding that there is an obvious "jump" phenomenon in the dissipation energy of surrounding rock during the process of gradually increasing displacement. A novel method for determining the optimum supporting opportunity of roadway based on energy dissipation was proposed, based on the above finding. This study can provide an original idea for the determination of roadway supporting opportunity.

## 1 Introduction

The tendency of looking for resources in the deeper parts of the Earth has been inevitable due to the depletion of shallow resources [1, 2]. The mineral resources is an important material support to guarantee the rapid growth of the whole society and economy, maintain the high quality of human survival and promote scientific and technological progress [3, 4], while safe and efficient mining technology is the crucial to ensure the normal operation of the resources extraction. Different from the condition environment of the shallow mineral resources, the surrounding rock in the deep condition presents "dangerous" characteristics of "Three High and One Disturbance" [5], once an incident occurs in the process of resources extraction, it will lead to a series of engineering accidents, and even more serious ones will result in great engineering disasters under the action of "disaster chain effect" [6–8]. Meanwhile, due to the special environment in the deep mining, it will bring significant difficulties to rescue activities and easily cause numbers casualties and property losses. Therefore, the stability control technology of deep mine is an important support for mineral resources extraction. The supporting time is too early, working zone is too close to the working face, which brings inconvenience to the construction, and the support structure is so overstressed that susceptible to damage; the supporting time is too late, the physical and mechanical properties of rock mass are excessively deteriorated, and the plastic zone of surrounding rock is malignant expansion, which leads to the overall collapse of roadway easily. The New Austrian Method [9] first proposed the concept of using the self-supporting capacity of surrounding rock to maintain roadway stability, and due to the New Austrian Method’s rationality and practicability, which has been widely applied in the engineering field. On this basis, He et al. [10] proposed the concept of the optimum supporting opportunity. The connotation is that the support is applied after the roadway has been excavated and before the rock relaxes, allowing a certain amount of deformation of the surrounding rock, but not so much that it develops to a detrimental degree. Therefore, constructing the supporting structure at the optimum supporting opportunity can not only give to the deformation control function of supporting adequately, but also improve the self-supporting capacity of surrounding rock, improve the stress state of surrounding rock, reduce the difficulty of supporting and save the cost of supporting.

Numerous researchers have investigated it from vary viewpoints to achieve the optimum supporting opportunity that is reasonable and supported by science. Wherein, the Convergence-Confinement method [11, 12] principle has been widely applied in the support design theory. It was first established in the 1960s of last century on the basis of the development of the New Austrian Method and active-support structure, and has been further improved with the application and popularization of field monitoring technology and numerical simulation software [13]. From the perspective of site monitoring and theory analysis, Kolymbas [14] reached the conclusion that large surrounding rock displacement should be avoided and enough construction space should be reserved before the initial support. Feng et al. [15] revealed the influence of support timing on the "surrounding rock-support" system based on field monitoring information, and proposed the support timing for the primary lining of surrounding rock based on this. Liu et al. [16] provided analytical solutions of tunnels in cold areas under different supporting opportunities and strengths. Choi et al. [17] obtained the best supporting opportunity of tunnel excavation in soft rock mass by theoretical analysis and numerical simulation according to the principle of New Austrian Method. Guan et al. [18] obtained the best support time of a typical tunnel section in Nagasaki County, Japan based on actual engineering sites by using the Burger-Deterioration model and deformation rate as the criterion. Peng et al. [19] introduced the rock mass failure proximity index (FAI) into the tunnel support design, defined the criterion of critical support time of surrounding rock, and established a method for determining the optimal support time of tunnel after reasonably considering the post-peak strain softening characteristics of rock mass based on the finite difference numerical calculation program. Jian et al. [20] established a mechanical model of the tunnel in view of the lack of reliable theoretical research on the timing of phased support for deep buried tunnels. Based on Mohr-Coulomb criterion, and considering the "spatial effect" of excavation, the aging characteristics of lining and the timing of phased application of supporting structure, The analytical solutions of plastic zone stress, wall displacement and support pressure during tunnel excavation and support are derived. Yan-jun et al. [13] established a corresponding three-dimensional numerical model based on the water delivery tunnel of a water supply project, adopted the finite difference method and Mohr-Coulomb yield criterion to conduct numerical simulation of construction excavation process, and studied the estimation of tunnel support opportunities under different surrounding rock conditions. Zhang et al. [21] put forward the approximate calculation formula of the optimal supporting time based on the new Austrian method and the aging deformation characteristics of underground engineering. Yong et al. [22] took the Niucheroof tunnel of Guang-wu Expressway as the engineering background and discussed the theoretical and numerical calculation method for determining the timing of the second lining support of the tunnel under the condition of rock mass rheology.

In terms of the stability control of deep roadway, a number of investigators have proposed active support, passive support, energy support, combined support, strong support, advanced support and stress control, etc., and these studies have promoted the development of roadway support technology [23]. Describing surrounding rock failure characteristics from the perspective of energy is one of the effective and essential methods to evaluate roadway stability. The energy support theory holds that surrounding rock failure is accompanied by energy dissipation, and supporting structure can absorb part of the energy. It is advocated to use support to control surrounding rock energy dissipation, so as to keep the roadway in a stable state [24, 25]. Compared with other theories, the energy theory considers the interaction between support and surrounding rock, which can better reveal the nature and control mechanism of roadway failure. Therefore, studying the energy dissipation evolution of surrounding rock has important reference significance for support design. Xie et al. [26, 27] analyzed the deformation and failure process of rock from the perspective of energy through a series of studies, revealed the energy dissipation and energy release characteristics of this process, and discussed the internal relationship between energy dissipation and energy release in the deformation and failure process of rock and rock strength and overall failure. Wang et al. [28] analyzed the influence of borehole pressure relief on roadway energy dissipation by means of numerical simulation, laboratory test and theoretical analysis. Yi et al. [29] studied the transfer and dissipation of strain energy in deep roadway surrounding rock under strain softening and dilatancy, and obtained some beneficial results. Based on the Mohr-Coulomb yield criterion and the generalized form of Hooke’s law, Gao et al. [30] derived the elastic strain-energy density equation of the element under three-dimensional stress state. On this basis, the energy absorption and release of surrounding rock during the evolution of coal reservoir roadway are quantitatively analyzed. These studies explain the nature of rock failure and further explore its principle from many aspects, which lays a foundation for the subsequent related research.

Currently, the greater part studies on the optimum supporting opportunity are based on the "Convergence-Confinement Method", which expounds and explores the optimum supporting opportunity from the macroscopic deformation of surrounding rock, while ignoring the essence of roadway deformation and failure. In a way, rock mass failure is an energy-driven instability of state. In this paper, based on energy dissipation theory, Fast Lagrangian Analysis of Continua in Three Dimensions (FLAC3D) is used to studied the influence of different supporting opportunities to surrounding rock energy dissipation, and try to put forward the determine method of optimum supporting opportunity from the perspective of energy, provided a novel idea for deep roadway support design.

## 2 Theoretical backgrounds

### 2.1 Convergence-confinement theory

**Fig 1:**
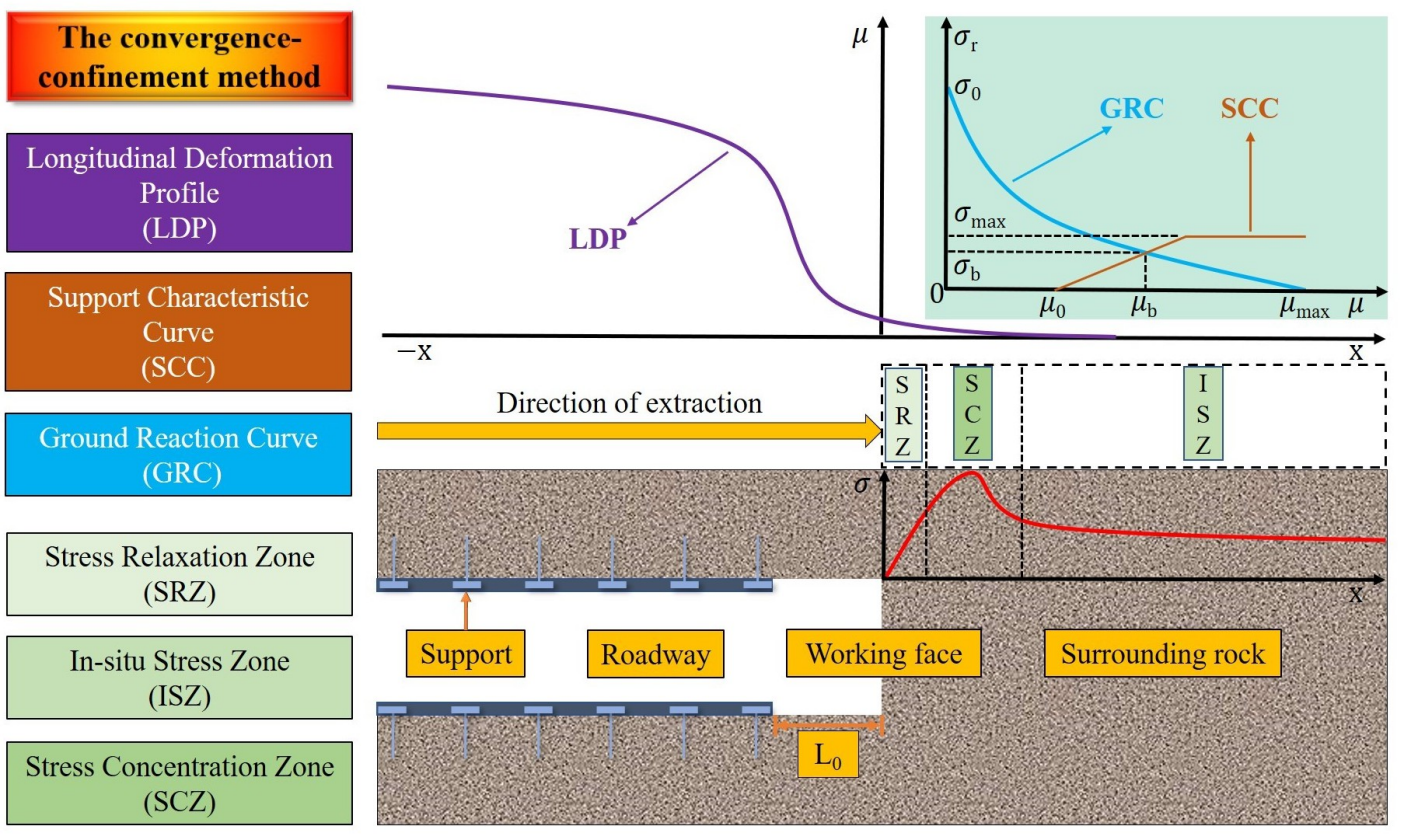
Schematic diagram of convergence-confinement method.

The Convergence-Confinement Method thought of surrounding rock and support structure was initially proposed by Fenner [31], which can effectively reflect the coupling between above structure during the roadway excavation [32]. The Convergence-Confinement method is composed of ground reaction curve (GRC), support characteristic curve (SCC) and longitudinal deformation profile (LDP), as shown in Fig 1. In the Fig 1, *σ*_0_ is the primary rock mass stress, *σ*_*s*_is the maximum supporting force, *σ*_*b*_ is the surrounding rock stress when the surrounding rock-supporting structure is in equilibrium, *μ*_0_ is the surface displacement of surrounding rock generated before support, *μ*_*b*_ is the displacement of surrounding rock when the surrounding rock-supporting structure is in equilibrium, and *μ*_*max*_ is the maximum displacement of surrounding rock without support. *L*_0_ is the distance between the supporting structure and the roadway driving face. *X* = 0 means it is at the working face; *x* ≤ 0 means it is behind the working face (not excavated area); *x* ≥ 0 means it is at the front of working face (excavated area). LDP reflects the convergence of surrounding rock displacement caused by roadway excavation near the working face. According to this curve, displacement still occurs in the unexcavated area. GRC reflects the relationship between the surrounding rock surface displacement and supporting force after roadway excavation. With the progress of stress redistribution, the surrounding rock displacement gradually increases, and the surrounding rock surface stress is gradually released. According to SCC, support stress gradually increases with the increase of surrounding rock displacement after the completion of support construction, and finally reaches the equilibrium state of surrounding rock and support structure [33]. There are three states of stress distribution behind the roadway working face, which can be divided into stress relaxation zone, stress concentration zone and in-situ stress zone according to the stress value, those state reflects the spatial characteristics of stress redistribution effect caused by roadway excavation.

**Fig 2:**
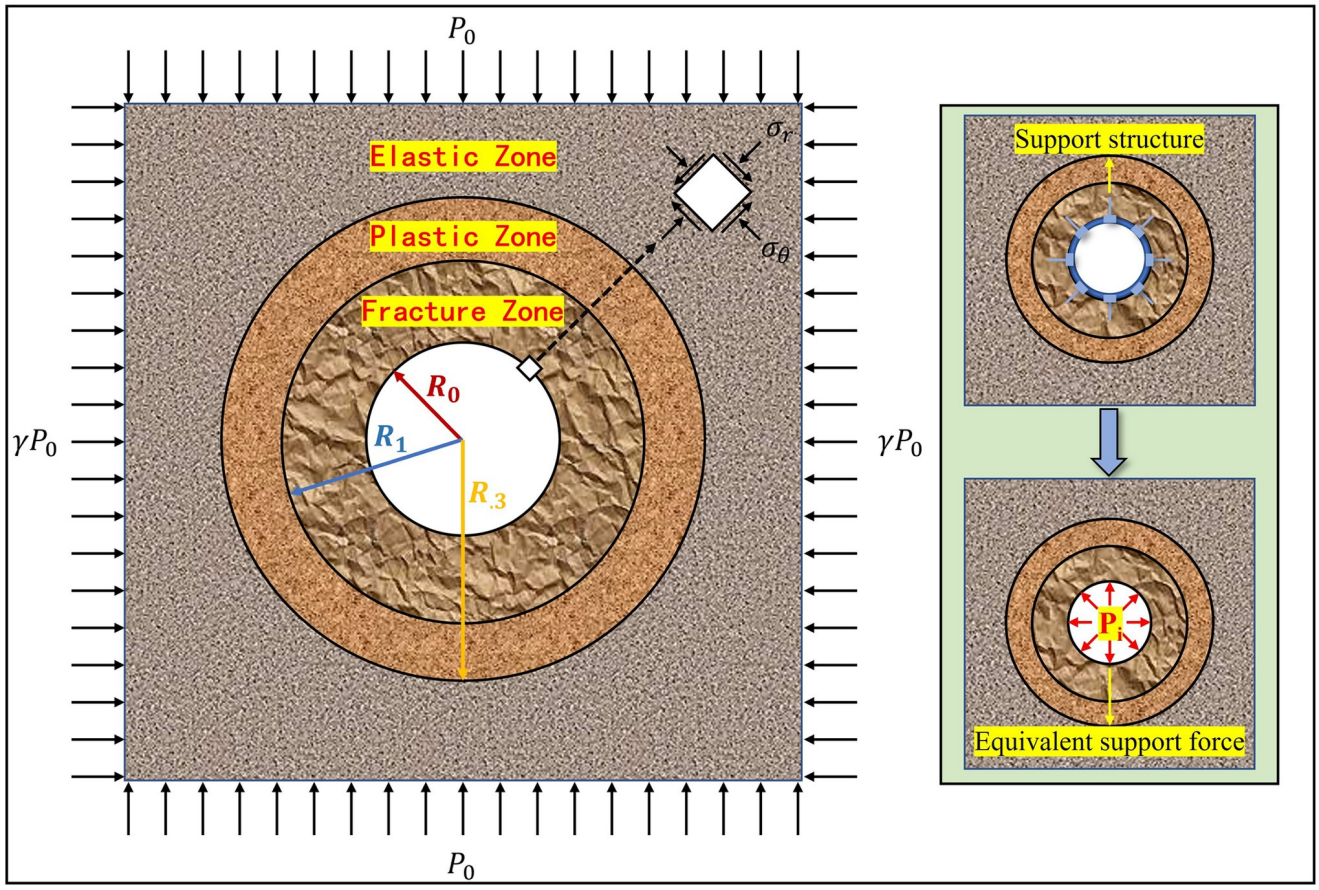
Mechanical model of roadway and equivalent supporting force of supporting structure.

This method has been widely recognized and applied in tunnel/roadway construction design, but it still has some limitations. For instance, the SCC is difficult to describe accurately. Although SCC has been obtained for supporting structures such as bolts/cables, shotcrete, lining and steel arche, the actual engineering support systems are complex, the roles played by the supports are different, and it is difficult to figure out the coupling mechanism of various support structures. Therefore, the acquisition of SCC accurately is still a challenge. Mechanical model of roadway and equivalent supporting force of supporting structure is shown in Fig 2.

### 2.2 Energy dissipation theory

**Fig 3:**
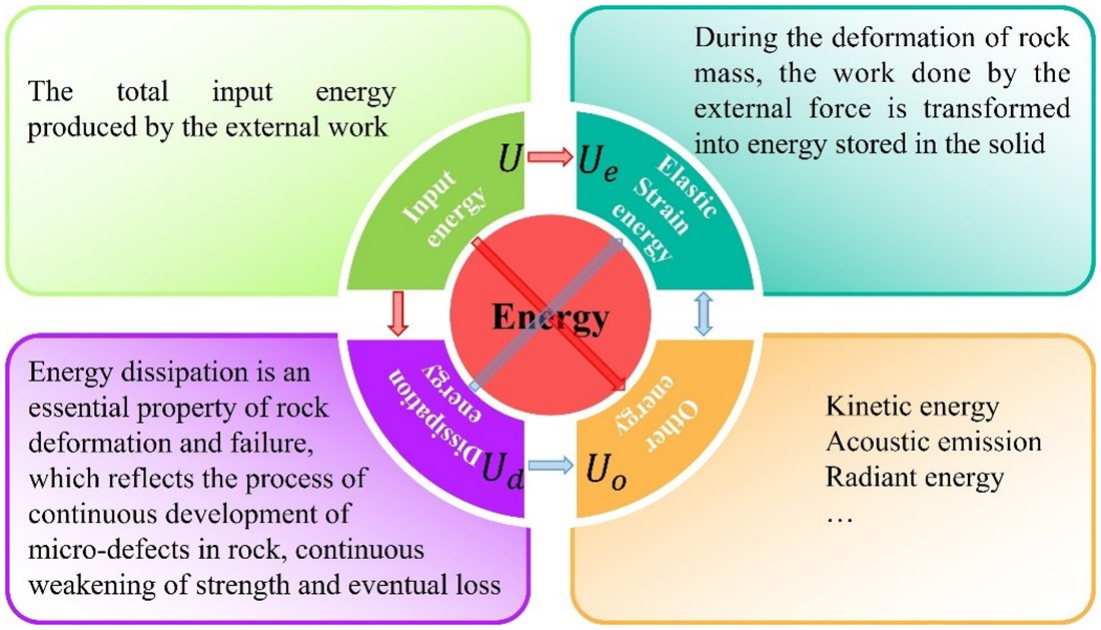
Various energy forms and interconversion relation in rock mass.

From an energy perspective, the essence of a roadway failure is the result of interconversion between different energy types. The essence of the phenomena such as rock fragmentation, side-roof collapse or rock explosion is a form of transformation between strain energy, dissipation energy, thermal energy, and kinetic energy. Moreover, kinetic energy and thermal energy are striking less than the strain energy and dissipation energy in rock mass. Therefore, the failure and deformation of roadway surrounding rock is dominantly caused by the release and dissipation of the energy (energy dissipation leads to the weaken of rock strength, and energy release leads to the disaster failure of rock mass) [34, 35]. Cook et al. [36–38] believe that when the energy released after the failure of the mechanical equilibrium between the orebody and the surrounding rock system is greater than the energy consumed, ore impact will occur. With more and more scholars studying rock mass from the perspective of energy, the energy theory in rock mechanics has made tremendous progress. Currently, it is believed that rock mass itself and its deformation process usually have various energy forms, such as elastic strain energy, plastic dissipated energy, surface dissipated energy, acoustic emission, radiation energy, kinetic energy and other energy forms [39], as shown in Fig 3.

Among them, some energies (such as radiant energy and dissipated energy, etc.) are extremely complicated to calculate, or it is difficult to get accurate results, and some energies are tiny and negligible compared with other energies. Xie [26] gave the following formula to describe the energy conversion rule in rock mass:

U=Ud+Ue (1)

Wherein, U is the total input energy generated by external work, U_d_ is the dissipated energy, and U_e_ is the elastic strain energy.


Ue=σ12+σ22+σ32-2νσ1σ2+σ2σ3+σ1σ32E (2)

Wherein, σ_1_, σ_2_, σ_3_ are the maximum principal stress, intermediate principal stress and minimum principal stress, respectively, ν is Poisson’s ratio and E is the elastic modulus.

**Fig 4:**
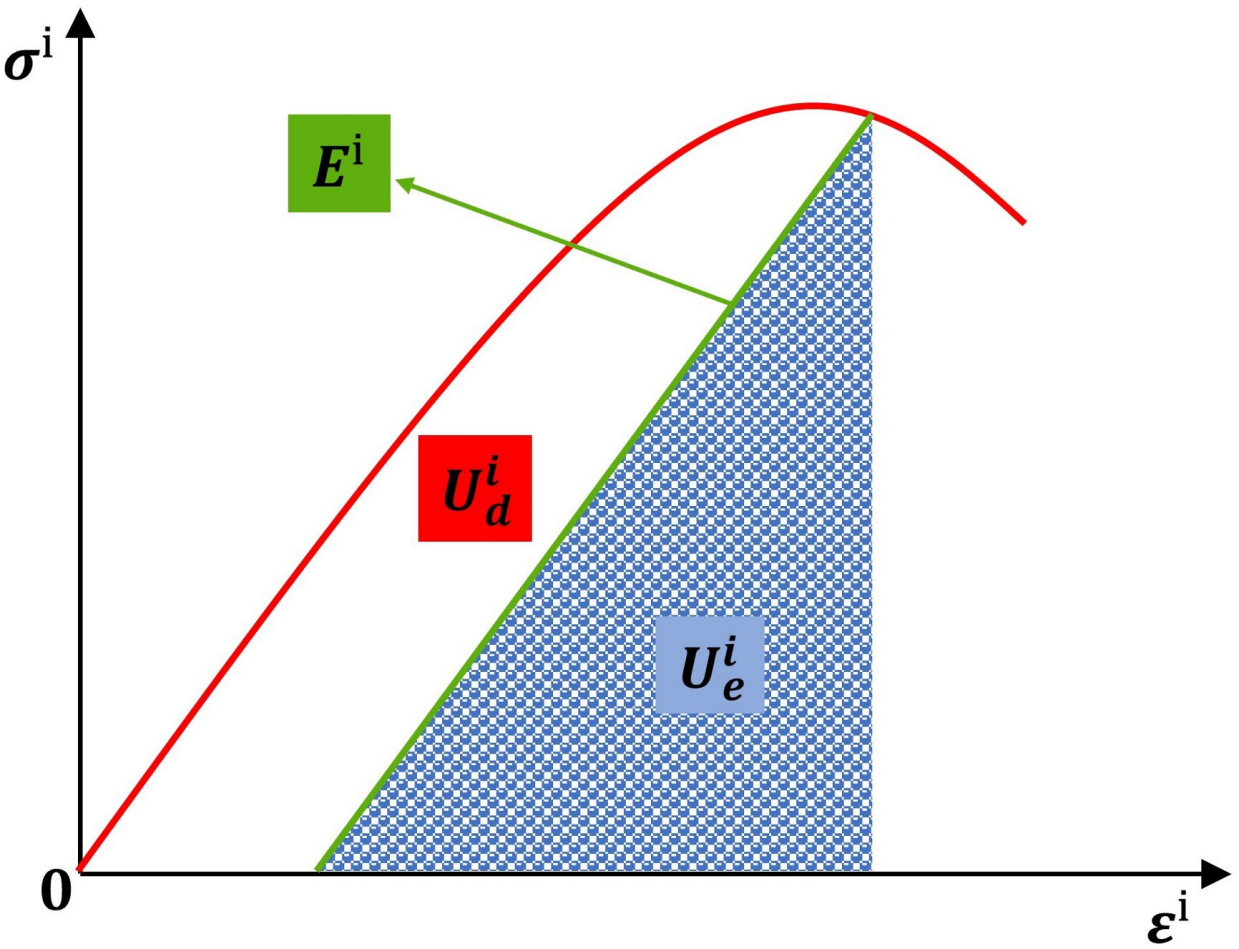
Relationship of energy release and energy dissipation [26].

Fig 4 shows the relationship of energy release and energy dissipation. The area $U_{d}^{i}$ represents the energy consumed when damage and plastic deformation occur in the unit, and the area $U_{e}^{i}$ represents the releable strain energy stored in the rock mass. The area E^i^ is the unloading elastic modulus.

### 2.3 Energy support theory

In 1979, Salamon and Wagner [40] firstly proposed the energy support theory. They believed that surrounding rock and support, as a coupling system, would interact and deform together. In the process of the surrounding rock-support system deformation, the supporting structure could absorb part of the energy released by surrounding rock, making the whole system in a steady state, the overall stability of the roadway is therefore maintained. This theory provides a reference for the follow-up study.

Energy dissipation will inevitably cause damage to rock mass, which will lead to the weaken of self-supporting capacity of surrounding rock. The accumulation of elastic strain energy will increase the probability of dynamic disaster. Based on this, from the perspective of energy dissipation and accumulation, attempts are made to connect the dissipation energy, which is difficult to be directly observed, with the macro displacement of roadway surrounding rock, explore the intrinsic connection between them, and try to propose a novel method to determine the optimum supporting opportunity of roadway. The two forms of energy are elaborated below:

1. As for the elastic strain energy, according to the research of Xie et al. [26], the following formula is used to express the elastic strain energy. In FLAC3D, the cloud map of elastic strain energy can be obtained by programming with Fish language program.
Ue=σ12+σ12+σ12-2νσ1σ2+σ2σ3+σ1σ32E (3)
2. Regarding the dissipation energy, according to the user help manual [41], the incremental solution program is used in the FLAC3D, that is, the equation of motion and stress-strain calculation are solved at each steps. There are a variety of plastic models available in FLAC3D that can be used to describe the deformation capacity of the region. When the region is irreversibly deformed, the energy is dissipated through plastic work. In addition, the strain in all region can be divided into elastic and plastic parts. The plastic part can be divided into volume dissipation energy and shear dissipation energy. The total shear energy change can be found from the total deviatoric strain and deviatoric stress:
ΔWTs=V2σ11+σ11'e11+σ22+σ22'e22+σ33+σ33'e33+2σ12+σ12'e12+2σ13+σ13'e13+2σ23+σ23'e23 (4)

The total volumetric change can be found from the total mean strain and mean stress:

ΔWTv=3V2σ¯-σ¯'e¯ (5)

The total shear plastic work dissipated during a step is the difference between the total shear energy and the elastic shear energy change at all steps:

ΔWps=ΔWTs-ΔWes (6)

Similarly, the total volumetric plastic work dissipated during a step.


ΔWpv=ΔWTv-ΔWev (7)

Each zone contains overlays of constant stress, strain tetrahedra. A volumetric average of the above energy components is calculated for the zone for an average energy dissipation increment for that zone. Each zones has four additional variables to accumulate dissipation energy:$W_{{pv}^{'}},W_{{ps}^{'}},W_{{ev}^{'}},W_{{es}^{'}}$.

The total plastic dissipation energy is the sum of shear plastic dissipation energy and volume plastic dissipation energy, which can be obtained by the built-in function of FLAC3D.

## 3. Numerical simulation verification

### 3.1 Model building

**Fig 5:**
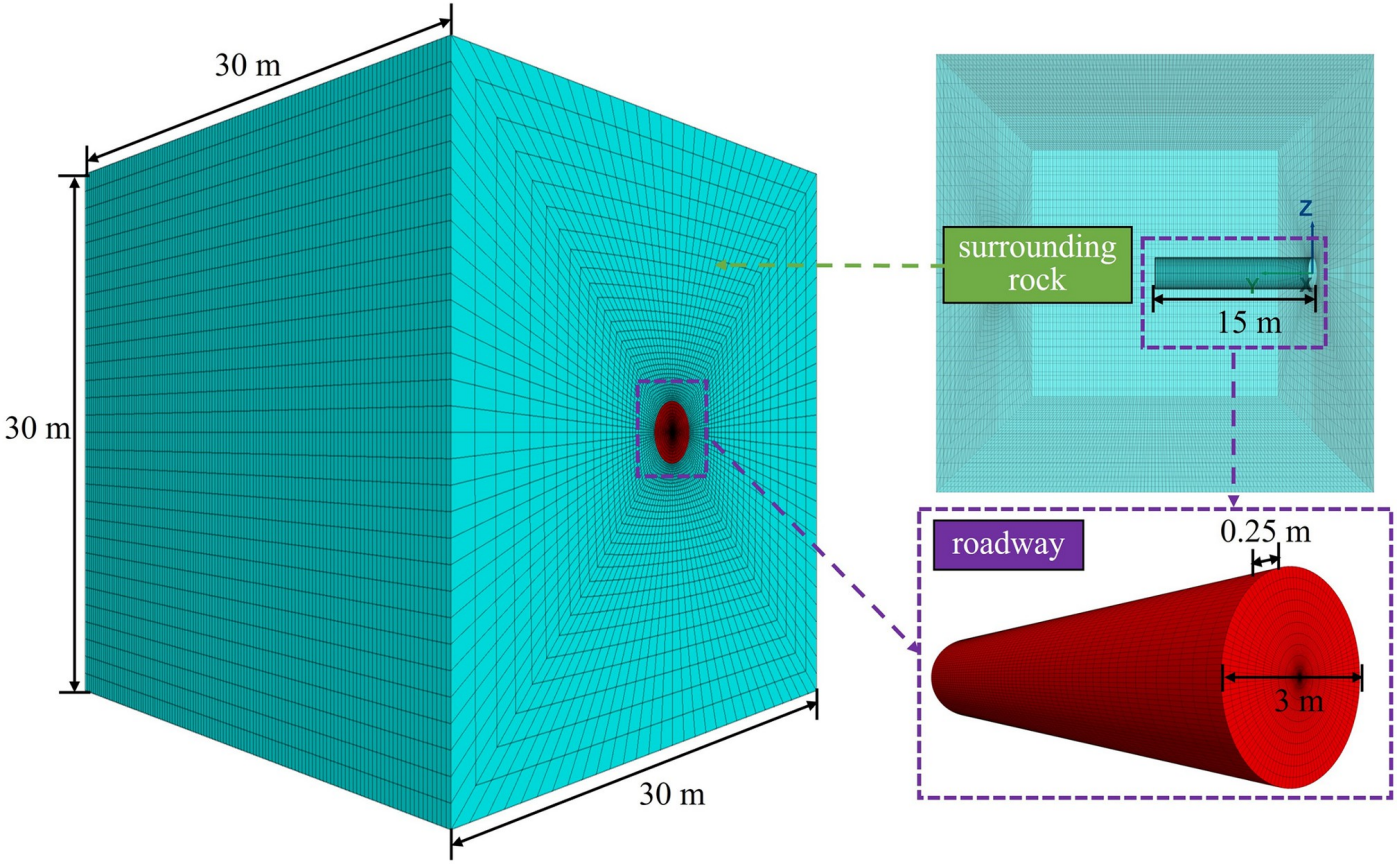
Three-dimensional numerical model.

**Fig 6:**
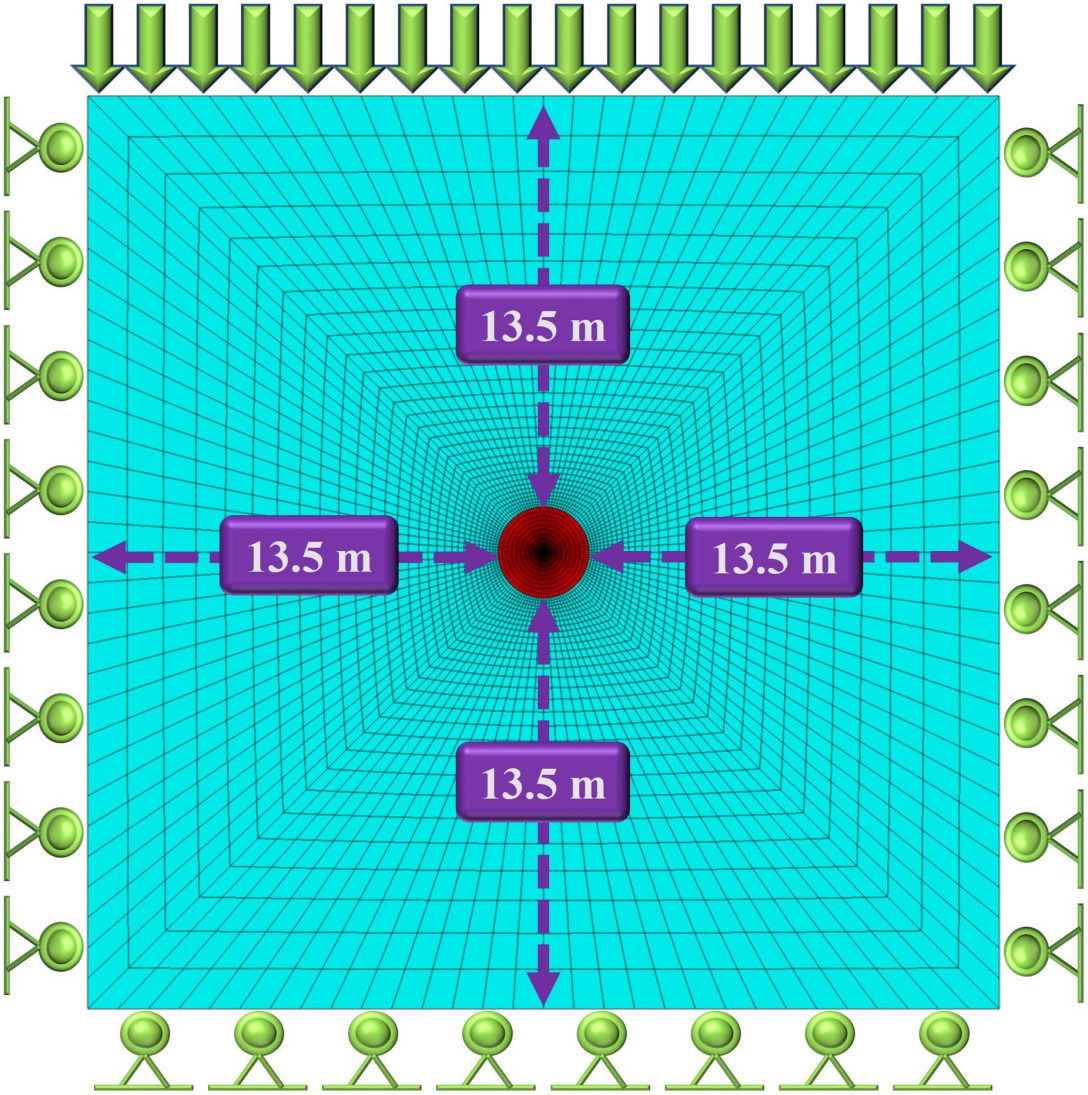
Model boundary condition.

Taking Sanshandao gold mine as an example and referring to the geology parameters in the literature [42], the cohesion and tensile strength in the original literature were selectively reduced to obtain more significant numerical simulation result. The model is assumed to have uniform geology condition and single lithology. Likewise, the vertical stress is 30 MPa and the lateral stress coefficient is 1.0, regardless of the self-gravity. The geology parameters of the model are shown in Table 1. A three-dimensional numerical model of the roadway and surrounding rock was established. The overall size of the model was 30 m × 30 m × 30 m. The section shape of the roadway is circular with a radius of 1.5 m. The top of model is free boundary, and 30 MPa uniform stress is applied above the model. The model has a total of 285,560 gridpoints and 288,000 zones. The Mohr-Coulomb provided in FLAC3D is selected as the constitutive model, which has been widely applied in geotechnical engineering and mining engineering. The schematic diagram of model and boundary conditions are shown in Figs 5 and 6.

**Table 1:** Geology parameters of the model.

Bulk modulus (GPa)	Shear modulus (GPa)	Internal friction angle (°)	Cohesion* (MPa)	Tensile strength* (MPa)
3.37	2.80	31	3.42(11.4)	1.31(4.37)

* Cohesion and tensile strength are decreased by 70% compared with the original.

### 3.2 Test scheme

**Fig 7:**
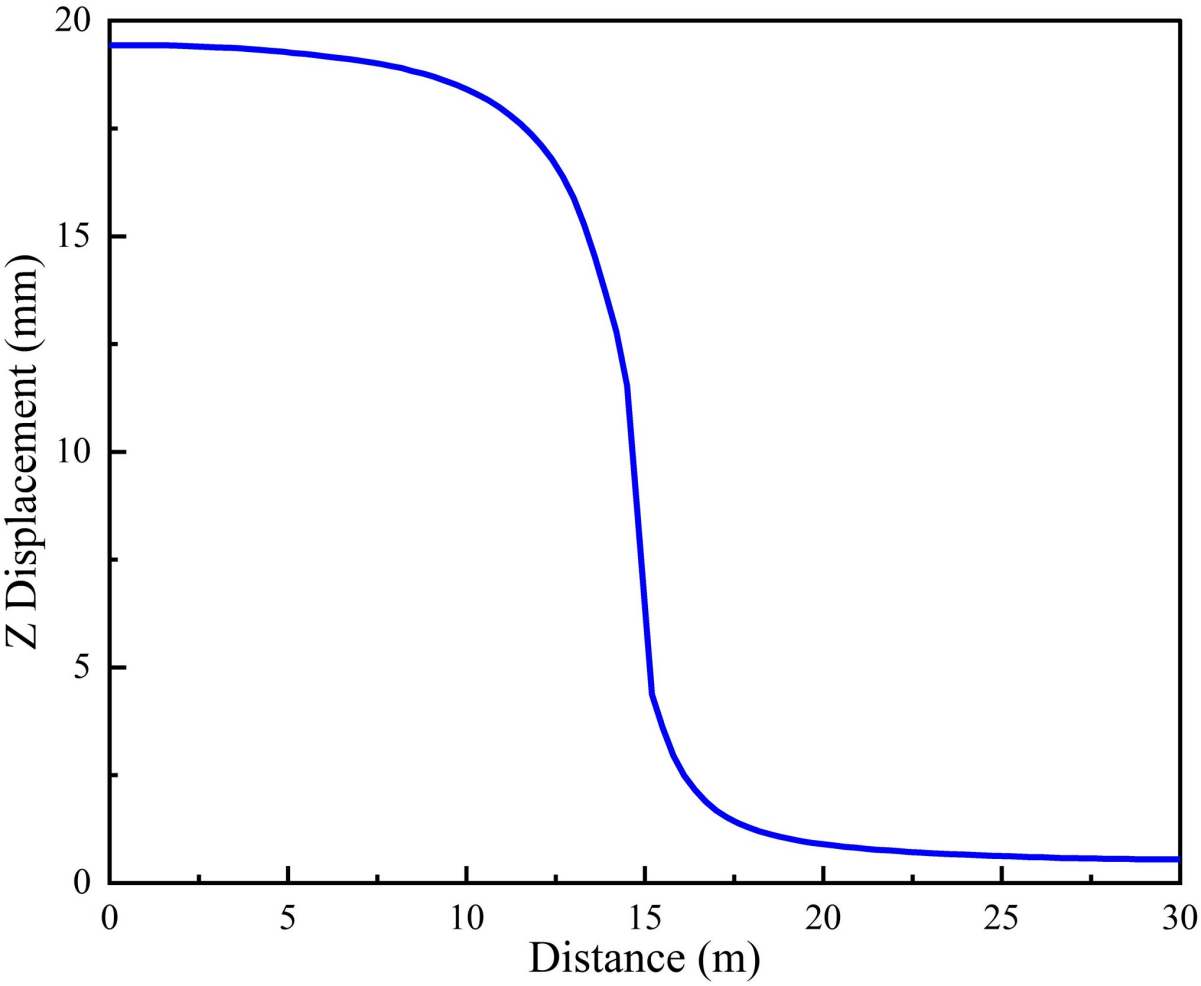
LDP.

**Fig 8:**
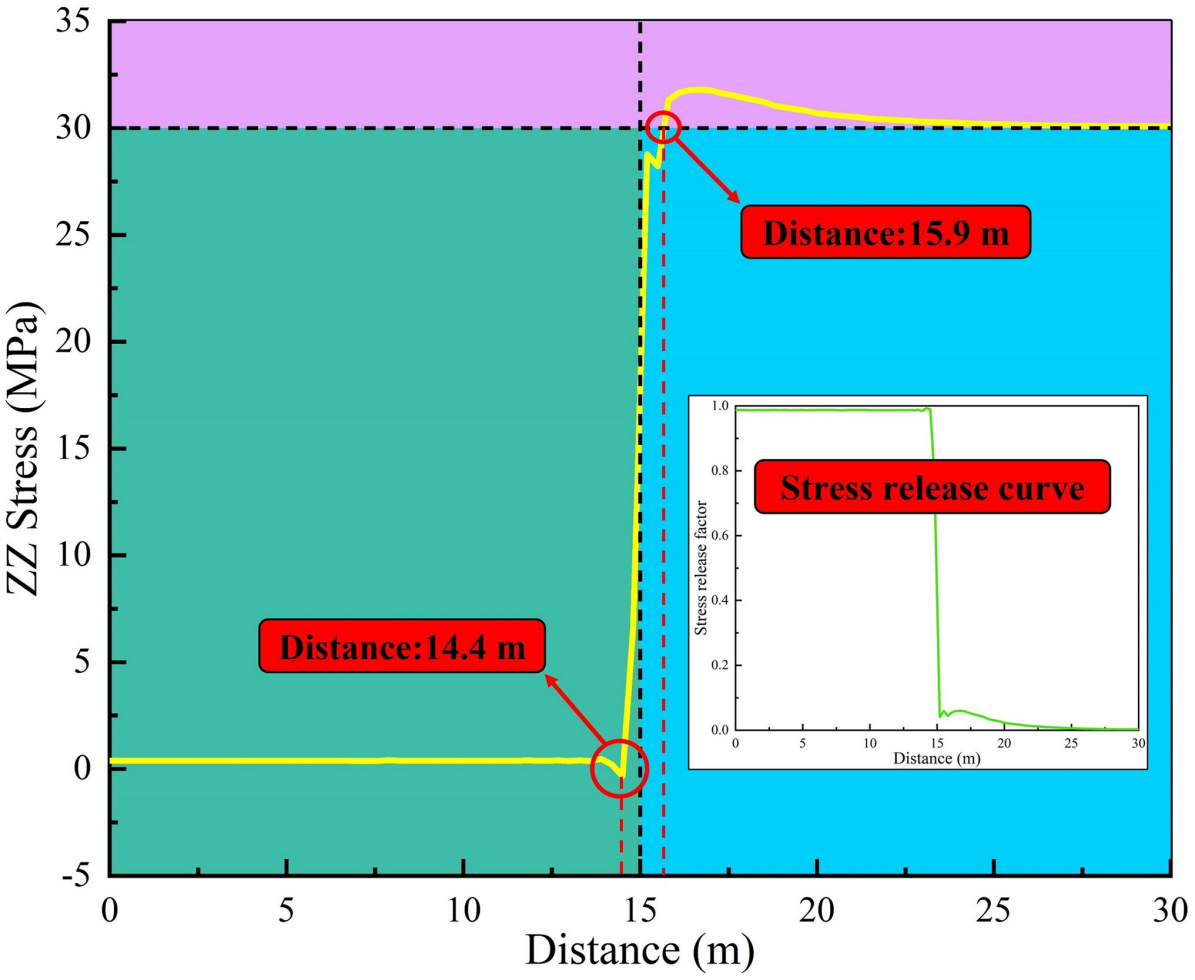
Stress curve.

**Fig 9:**
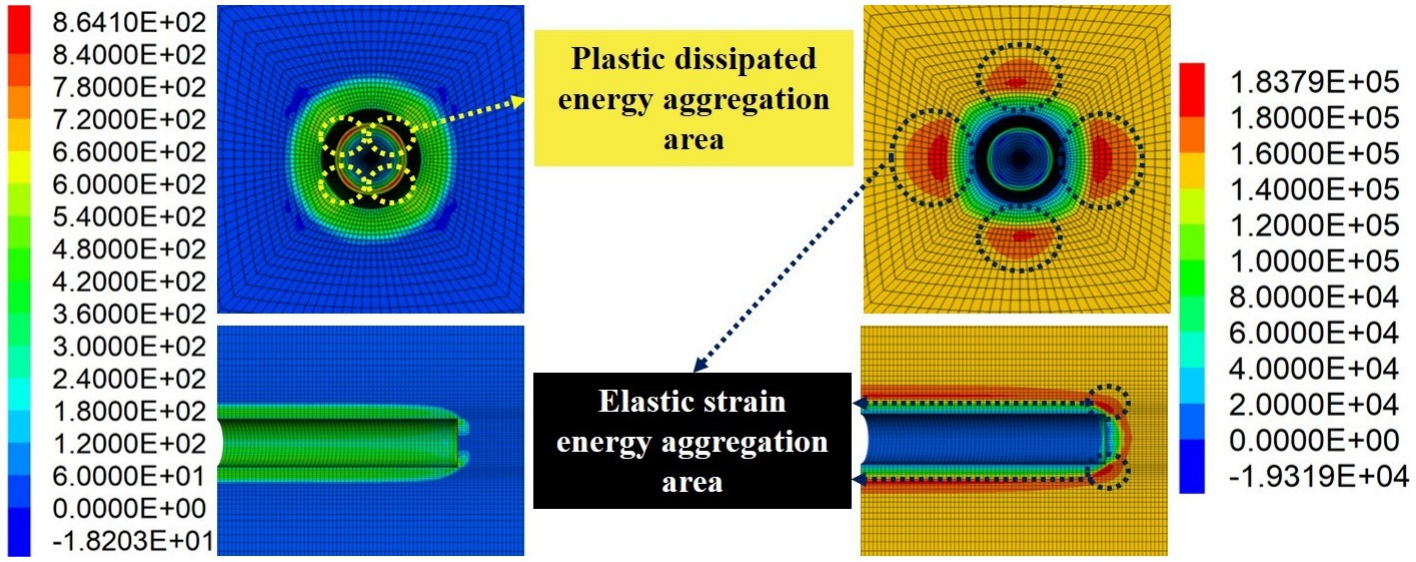
Energy cloud diagram (Unit: J). (a) Plastic dissipated energy, (b) Elastic strain energy.

Support opportunity mainly includes two dimensions of time and space, namely the apply time and the apply position of surrounding rock support, which will directly affect the energy evolution law of rock mass. Firstly, the roadway excavation simulation without support was carried out to obtain LDP and vertical stress curves of the roadway under this condition, as shown in Figs 7 and 8. According to Fig 7, the vertical displacement of the roadway roof has basically reached the maximum value (19 mm) at the 0 m to 10 m area, and the value of displacement decreases sharply at the 10 m to 15 m area, indicating that the confinement effect of the working face is taking effect. The displacement at the 10 m to 15 m area is tiny, indicating that the the confinement effect of the working face is limited. At the 20 m to 30 m area, displacement close to nil, indicating that this area has been basically unaffected by excavation. Fig 8 shows the roadway excavation leads to the redistribution of stress in the surrounding rock, and the stress near the working face changes significantly. At 0.6 m from the working face, the stress is basically released completely (calculated by the model to the maximum unbalance rate to 1e-5). Fig 9 represents the energy cloud diagram in the model. The elastic strain energy mainly accumulates around the roadway about 1 m away from the surface of the roadway and 1 m directly behind the excavation face. In addition, the elastic energy accumulates around the excavation face edge 0.3 m, with the maximum value up to 183.8 KJ. In the circular roadway, rock-burst therefore mainly comes from these parts. The dissipation energy mainly exists in the four corners of the working face edge, with the maximum value up to 864.1 J, and there is basically no energy dissipation in the working face center.

**Fig 10:**
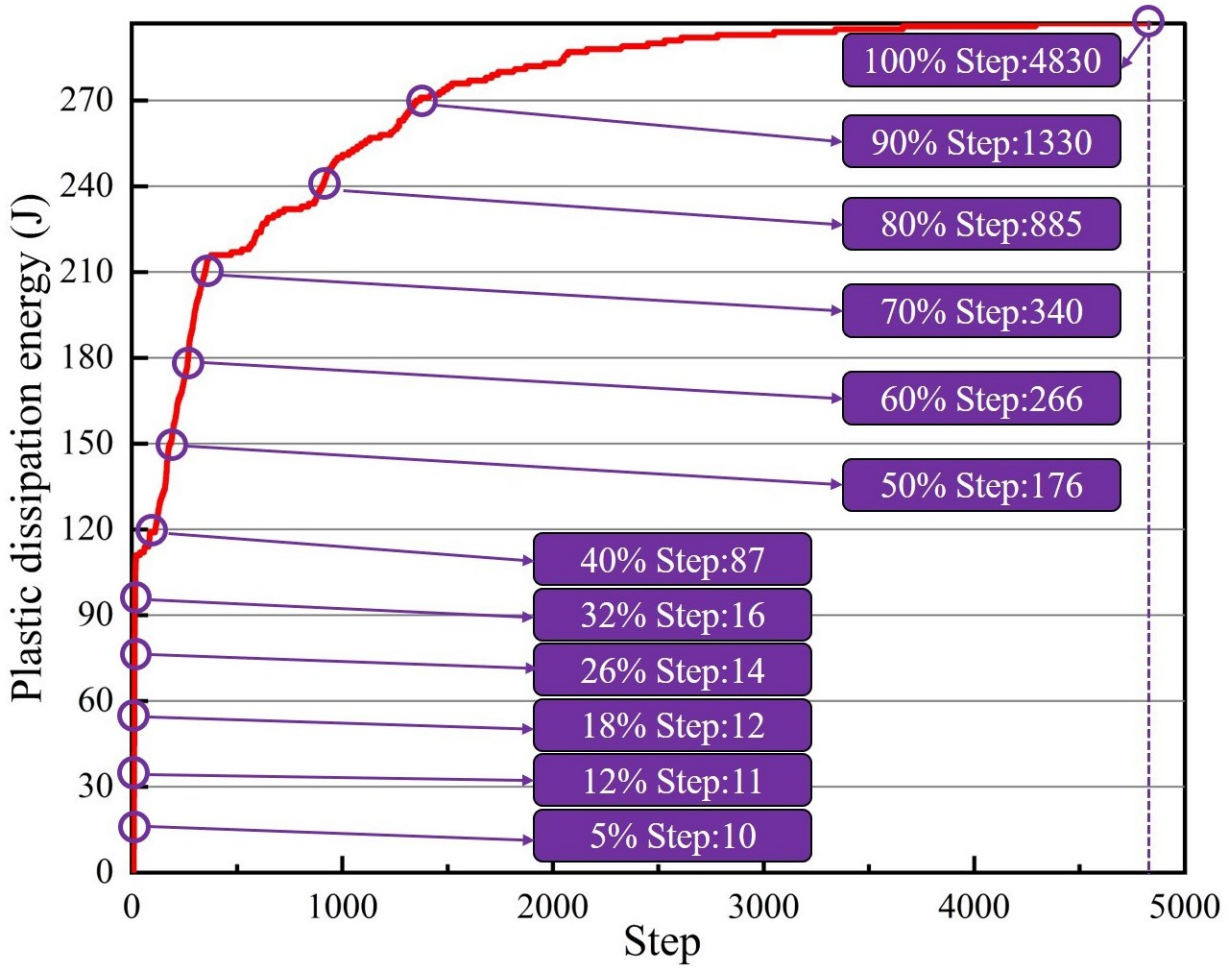
Supporting time scheme.

**Fig 11:**
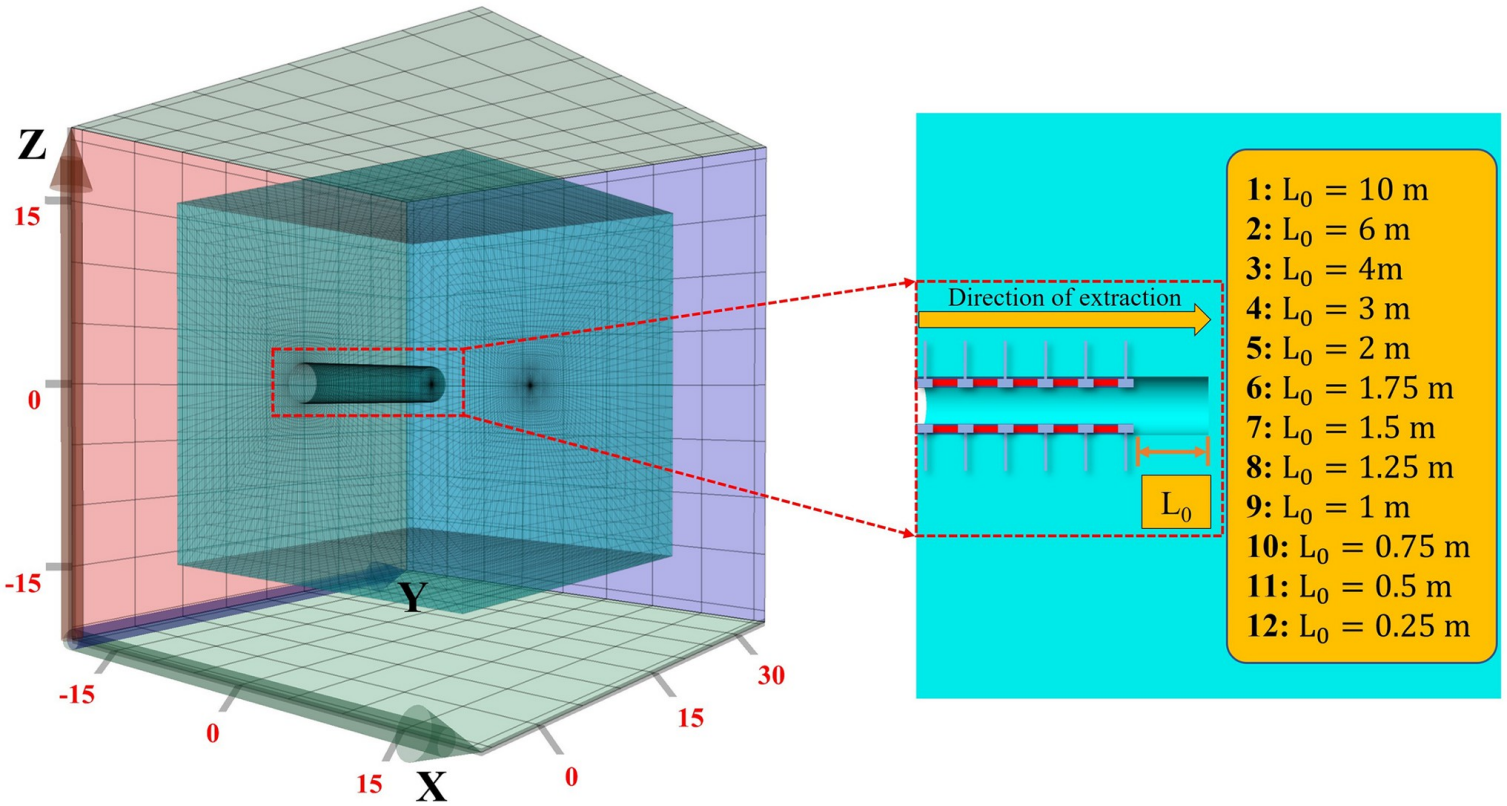
Supporting position scheme.

In FLAC3D, the calculation step in non-dynamic mode has no relationship with the real-time, but the evolution law of model is valuable. Fig 10 records the energy dissipation law at the center of the roof front end surface of roadway excavation process. Baes on the curve, the growth process of plastic dissipated energy of the roadway surrounding rock was divided into twelve stages (the dissipated energy generated at each step in the simulation process was not uniform, and the jump in the early stage was large). The values of each process are 5%, 12%, 18%, 26%, 32%, 40%, 50%, 60%, 70%, 80%, 90% and 100% of the maximum plastic dissipated energy at the point, respectively. According to the principle of single variable, combined with the experience in field practice and Fig 11, the control support position was selected as 2 m away from the roadway working face.

Twelve schemes are proposed to be 0.25 m, 0.5 m, 0.75 m, 1.0 m, 1.25 m, 1.5 m, 1.75 m, 2.0 m, 3 m, 4 m, 6 m, and 10 m away from the roadway working face, as shown in Fig 11. Referring to energy dissipation history curve, the calculation step when it is 40% of the maximum plastic dissipated energy of roadway surrounding rock is selected, that is, step = 87. When determining the support position, the monitoring position of energy dissipation is the center of the excavation face roof surface.

In this study, according to the relevant calculation formula in the literature [43], the maximum support resistance of the combination of steel bracket and anchor mesh was calculated to be 1.34 MPa, and the maximum support resistance of anchor rods was 0.134 MPa. Considering the possible loss of the support effect, the equivalent support force was taken to be 1 MPa and uniformly applied to the inner wall surface of the roadway.

### 3.3 Results

#### 3.3.1 Plastic zone

**Fig 12:**
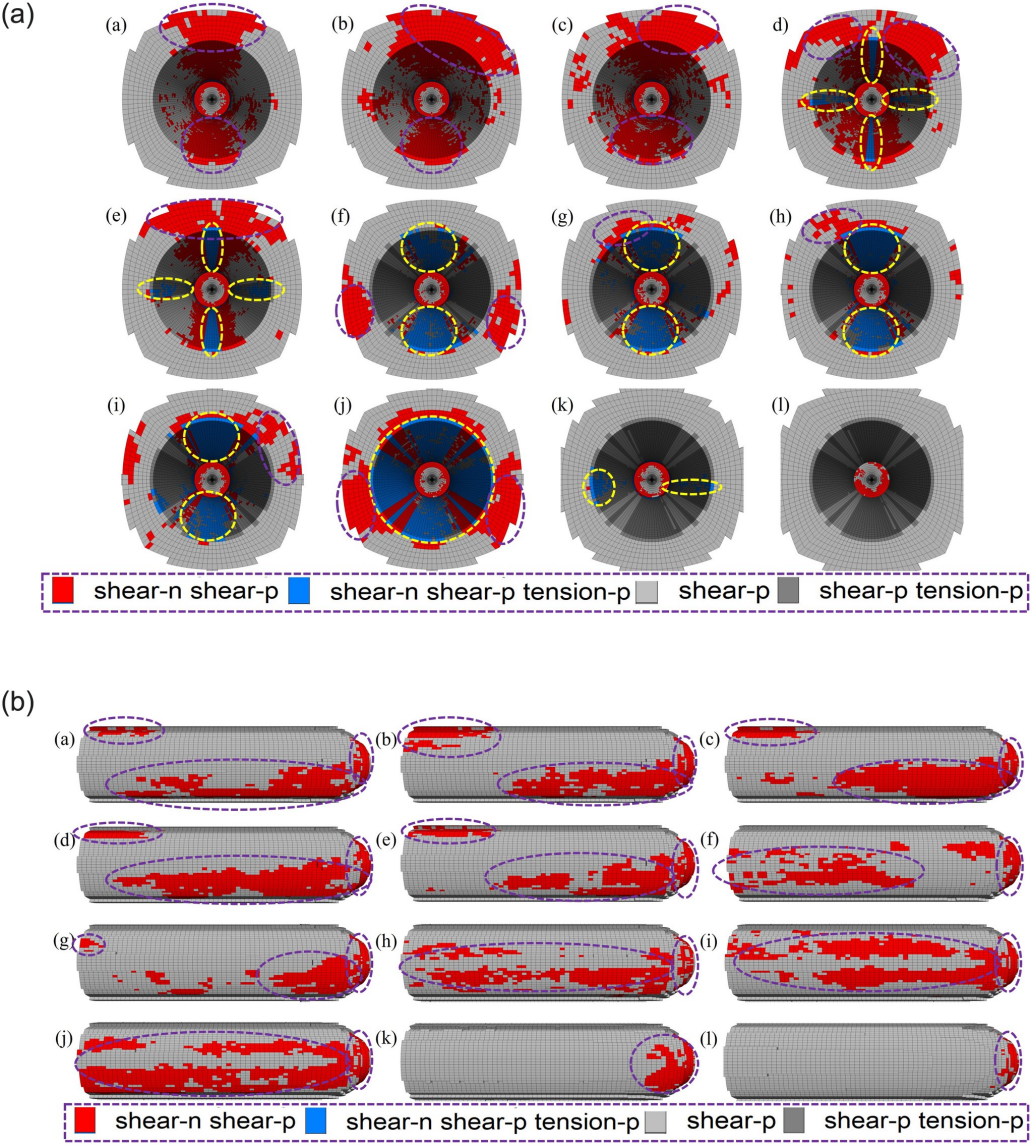
State distribution of plastic zone in different supporting time schemes. (a) front, (b) side.

**Fig 13:**
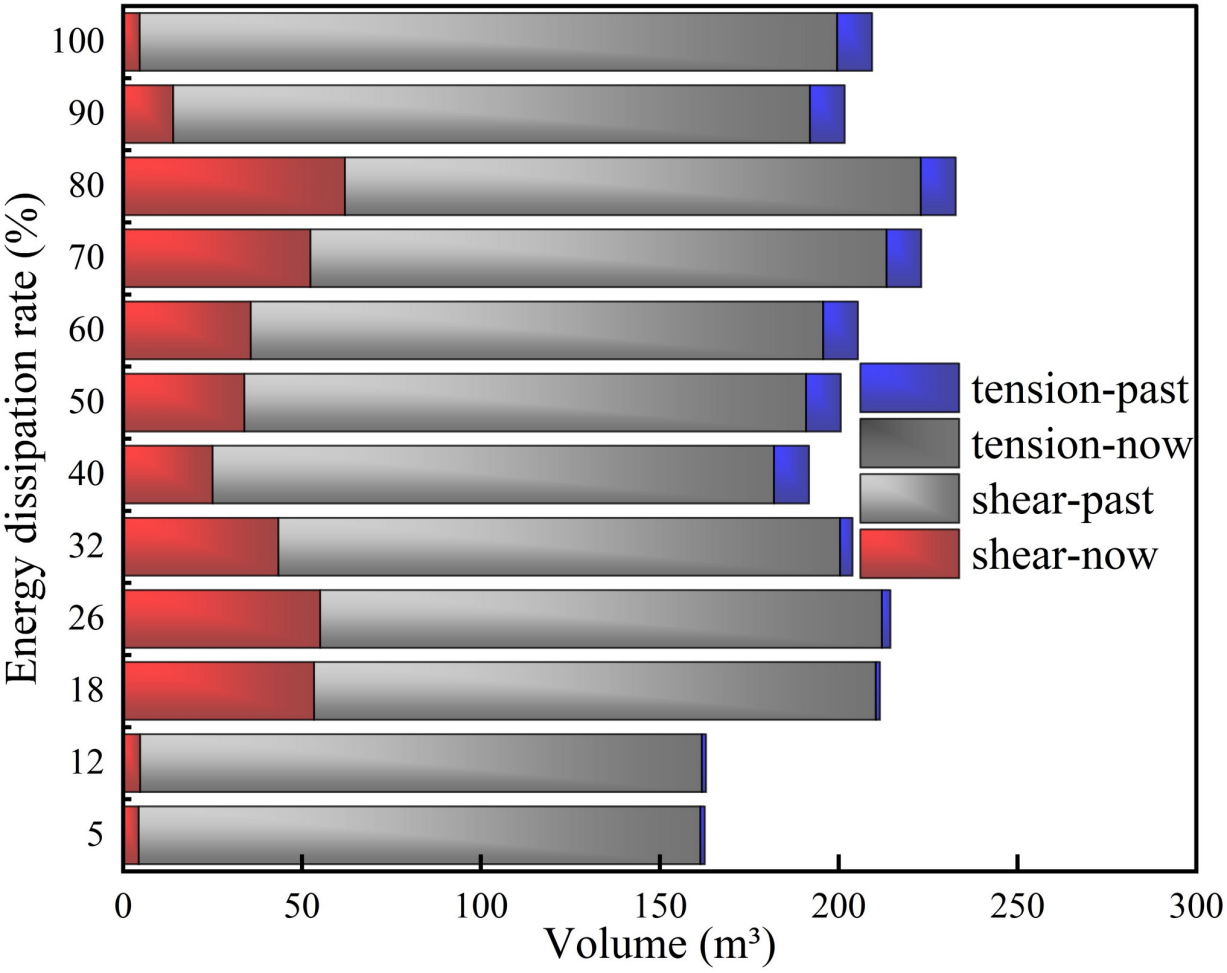
Plastic zone volume in different supporting time schemes.

*(1) Supporting time*. Figs 12 and 13 show the model plastic zone distribution characteristics, plastic zone volume statistics and results for different supporting time schemes, respectively. According to Fig 12 that all the existing plastic zones of the roadway are shear failure plastic zones, and under all support schemes, plastic shear failure zones exist at the edge of the working face, while tensile failure has occurred in a few areas, all of which exist on the inner surface of the roadway. When the energy dissipation ratio reaches 5%, 12%, 18%, 24% and 32%, the existing shear plastic zone mainly exists at the front end and the back side of the roadway. When the energy dissipation ratio reaches 40%, the existing shear failure zone at the front end of the roadway disappears and is mainly distributed at the two sides of the roadway. The upper and lower surfaces of the inner wall of the roadway also begin to appear areas that have sent shear and tensile failure at the same time in the past and have existing shear failure. When the energy dissipation ratio is above 90% for support, the existing plastic zone of the roadway gradually decreases and mainly concentrates behind the roadway working face. According to Fig 13, as the energy dissipation ratio increases gradually, the plastic zone volume presents the characteristics of "climb up and then decline". When the energy dissipation ratio reaches 40%, the overall plastic zone is relatively less, 5% < 12% < 100% < 90% < 40% < others.

**Fig 14:**
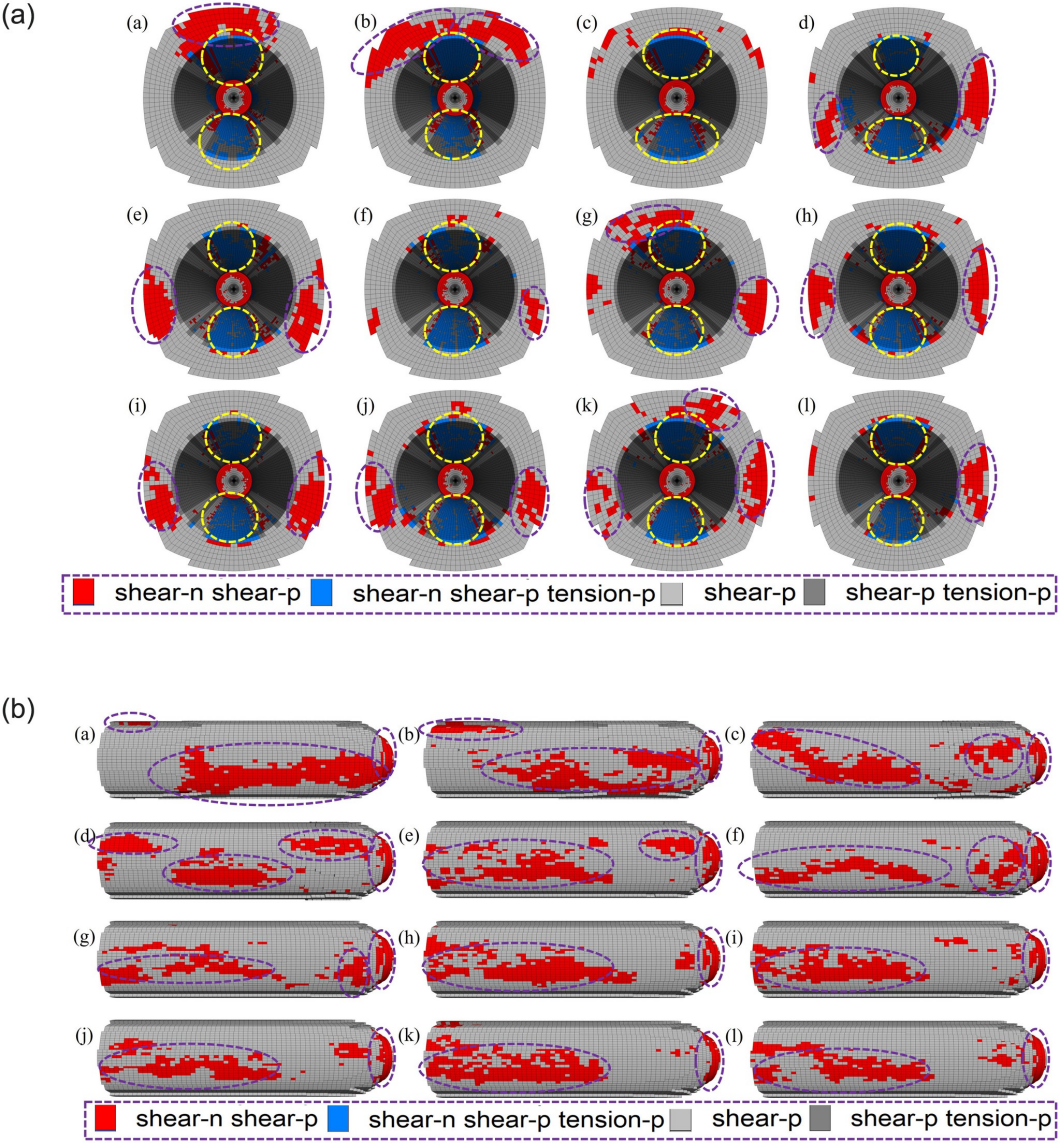
State distribution of plastic zone in different supporting position schemes. (a) front, (b) side.

**Fig 15:**
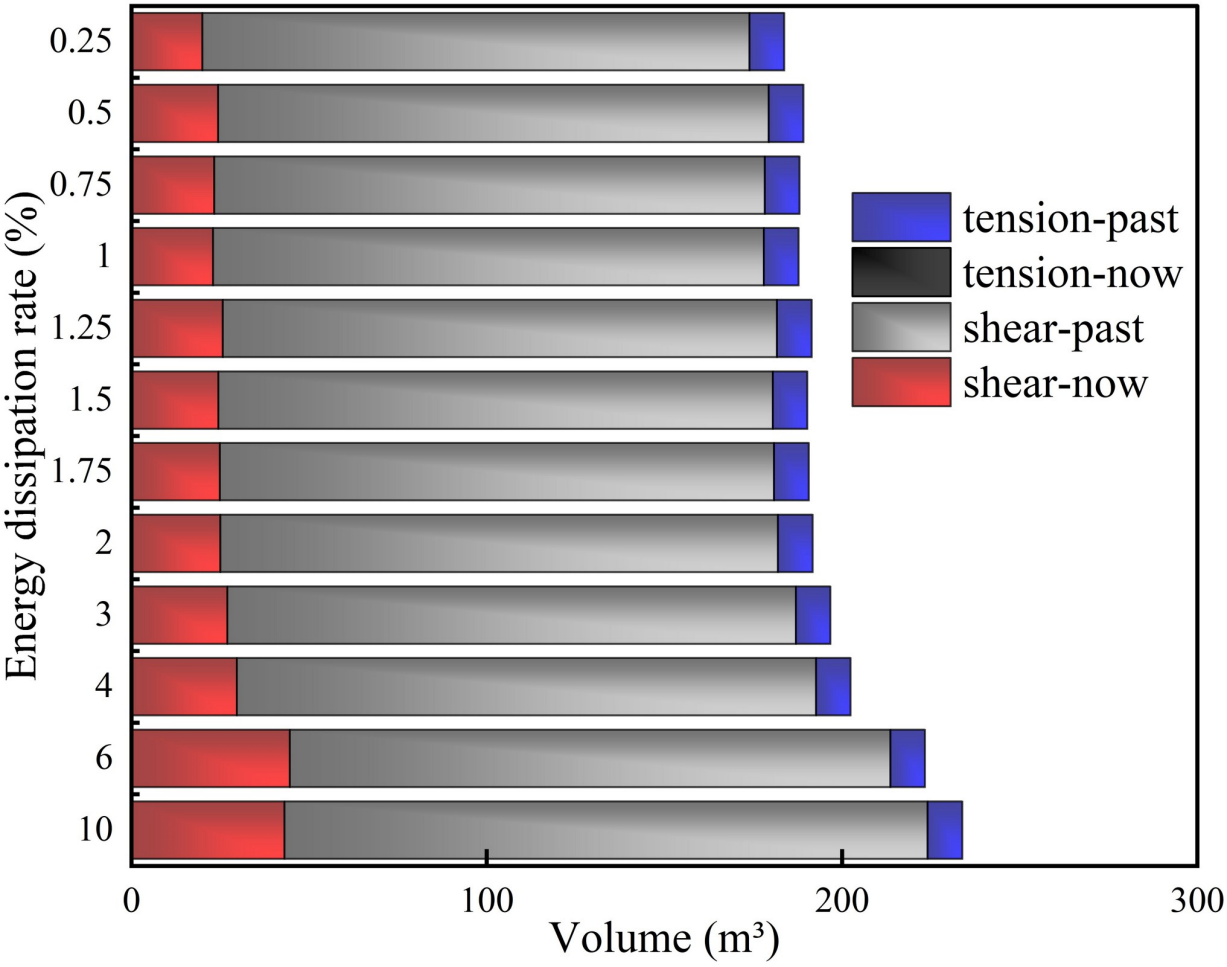
Plastic zone volumes in different supporting position schemes.

*(2) Supporting position*. Figs 14 and 15 show the model plastic zone distribution characteristics, plastic zone volume statistics and results for different supporting position schemes, respectively. According to Fig 14, all the existing plastic zones of the roadway are shear failure plastic zones, and under all supporting position schemes, plastic shear failure zones exist at the edge of the working face, while tensile failure has occurred in a few numbers of areas, all of which exist on the inner surface of the roadway. When the support is 10 m, 6 m, 4 m, 3 m away from the working face, there is a shear failure plastic zone at the front end of the roadway excavation. When the support is 2 m away from the working face, the existing plastic zone begins to be mainly distributed in the two sides of the front end of the roadway, and the total volume of the plastic zone decreases. According to Fig 15, as the supporting structure gradually approaches the excavation face, the plastic zone volume presents a "decline" feature. When the distance from the working face is 2 m, the decline trend of the plastic zone volume slows down.

#### 3.3.2 Displacement

**Fig 16:**
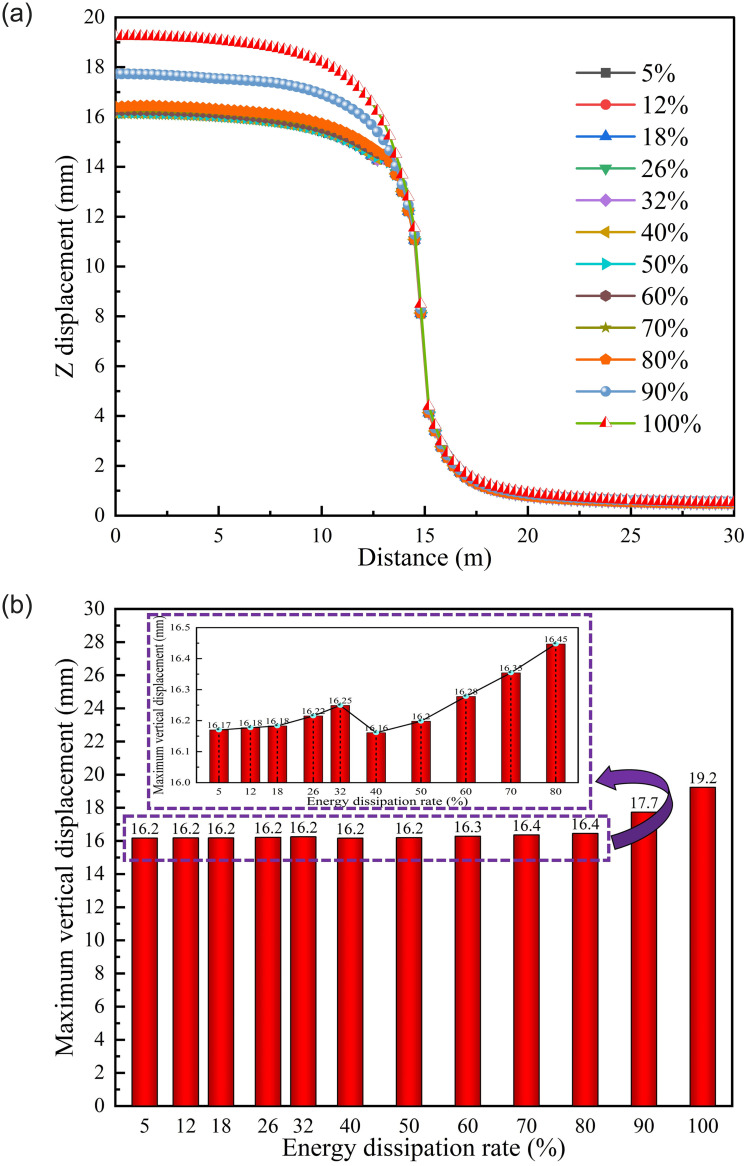
Roadway displacement of different supporting time schemes. (a) LDP, (b) Maximum displacement correlation.

*(1) Supporting time*. Fig 16 shows the displacement of roadway in different supporting time schemes. According to Fig 16 that when energy dissipation reaches 90% and 100% for support, the displacement will be significantly larger than that in other schemes. Under different supporting time schemes, the maximum displacement of the roadway presents a trend of "first increase, then decrease and then increase". When the energy dissipation reaches 40%, the maximum displacement of the roadway is 16.16 mm, which is the minimum displacement in all schemes. Subsequently, the maximum displacement of the roadway gradually increases.

**Fig 17:**
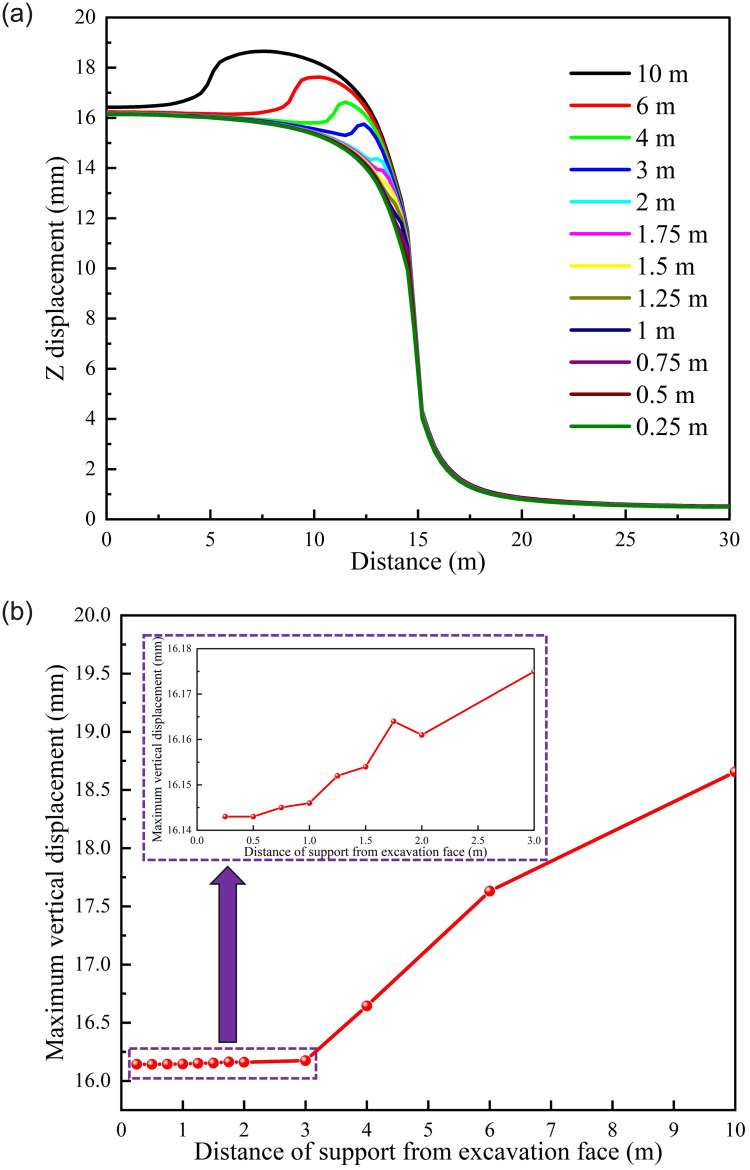
Tunnel displacement of different support position schemes. (a) LDP, (b) Maximum displacement correlation.

*(2) Supporting position*. Fig 17 shows the displacement of different supporting position schemes. According to Fig 17(a), LDP is significantly correlated with supporting position, different from the roadway displacement characteristics of different supporting time schemes. The displacement of areas with support is significantly lower than that of areas without support. The maximum displacement of the roadway is 18.656 mm. According to Fig 17(b) that the maximum displacement of the roadway under different supporting position schemes shows an "increasing" trend roughly. When the supporting position of the roadway is within 3 m, the maximum displacement is significantly lower than that when the supporting position is outside 3 m, and 0.25 m is the minimum displacement in all schemes.

#### 3.3.3 Energy

**Fig 18:**
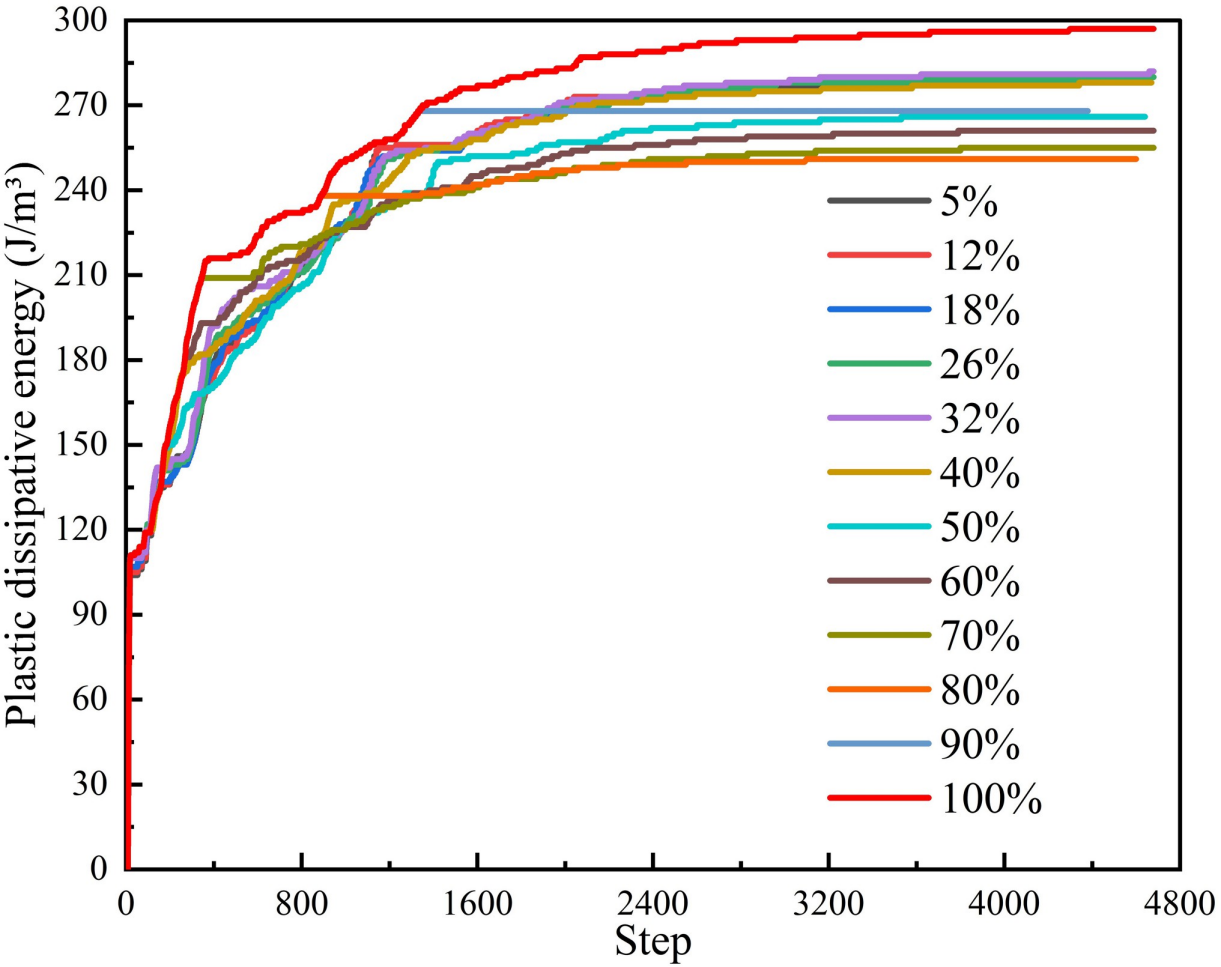
Energy dissipation curves of different supporting time schemes.

**Fig 19:**
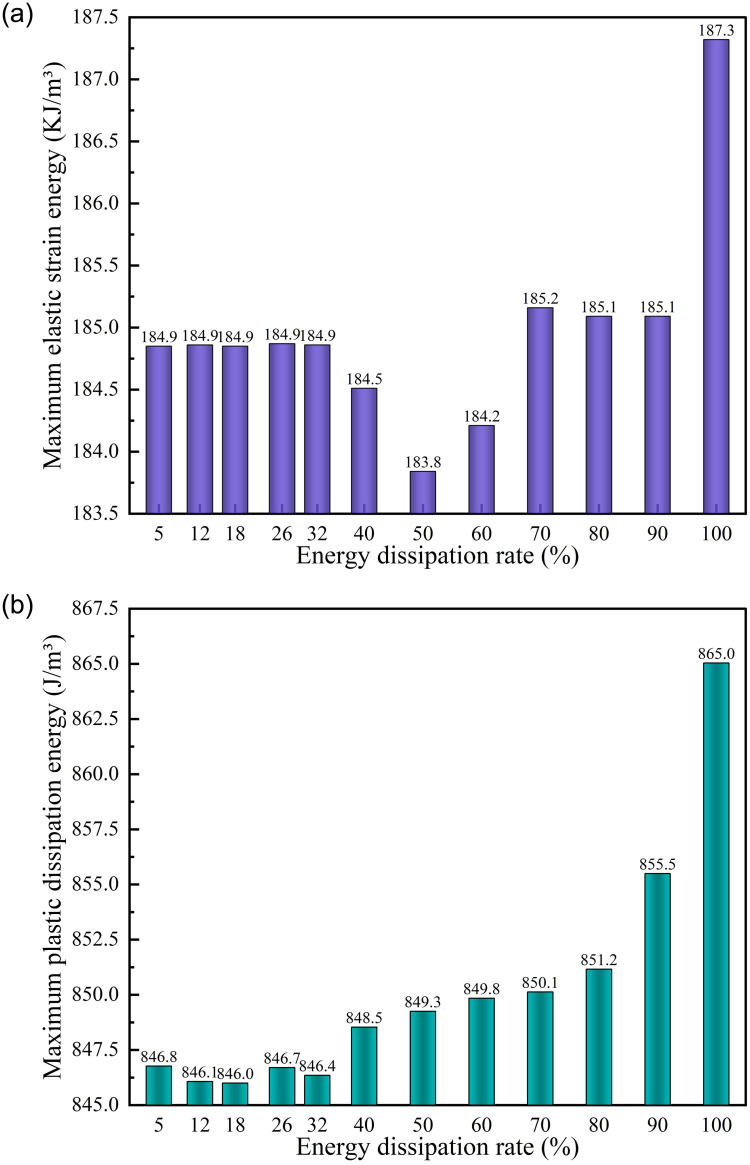
Energy dissipation of different supporting time schemes. (a) Maximum elastic strain energy, (b) Maximum plastic dissipation energy.

*(1) Supporting time*. Under different supporting time schemes, energy dissipation monitoring was conducted on the central position of the upper surface of the initial excavation position of the roadway, as shown in Fig 18. According to Fig 18 that the plastic dissipated energy rapidly increased to about 40% after excavation, and then the growth rate gradually decreased. When the energy dissipation reached about 90%, the growth rate became extremely slow. From the overall energy dissipation value, 100% > 32% > 26% > 18% > 12% > 5% > 40% >90% > 50% > 60% >70% > 80%, among which 5%, 18%, 26% and 32% have little difference in the whole, showing a noteworthy degree of energy dissipation. The maximum energy dissipation of different supporting time schemes in the model is shown in Fig 19. According to Fig 19(a), when the plastic dissipation energy reaches 100%, the maximum elastic strain energy will be significantly greater than that of other supporting schemes. The lowest is 183.8 KJ at 50%, and the highest is 187.3 KJ at 100%. According to Fig 19(b), the maximum dissipation energy generally presents a "flattening-increasing" trend with the passing of supporting time, and a sudden increase occurs when the plastic dissipated energy reaches 40%, 90% and 100% of the schemes.

**Fig 20:**
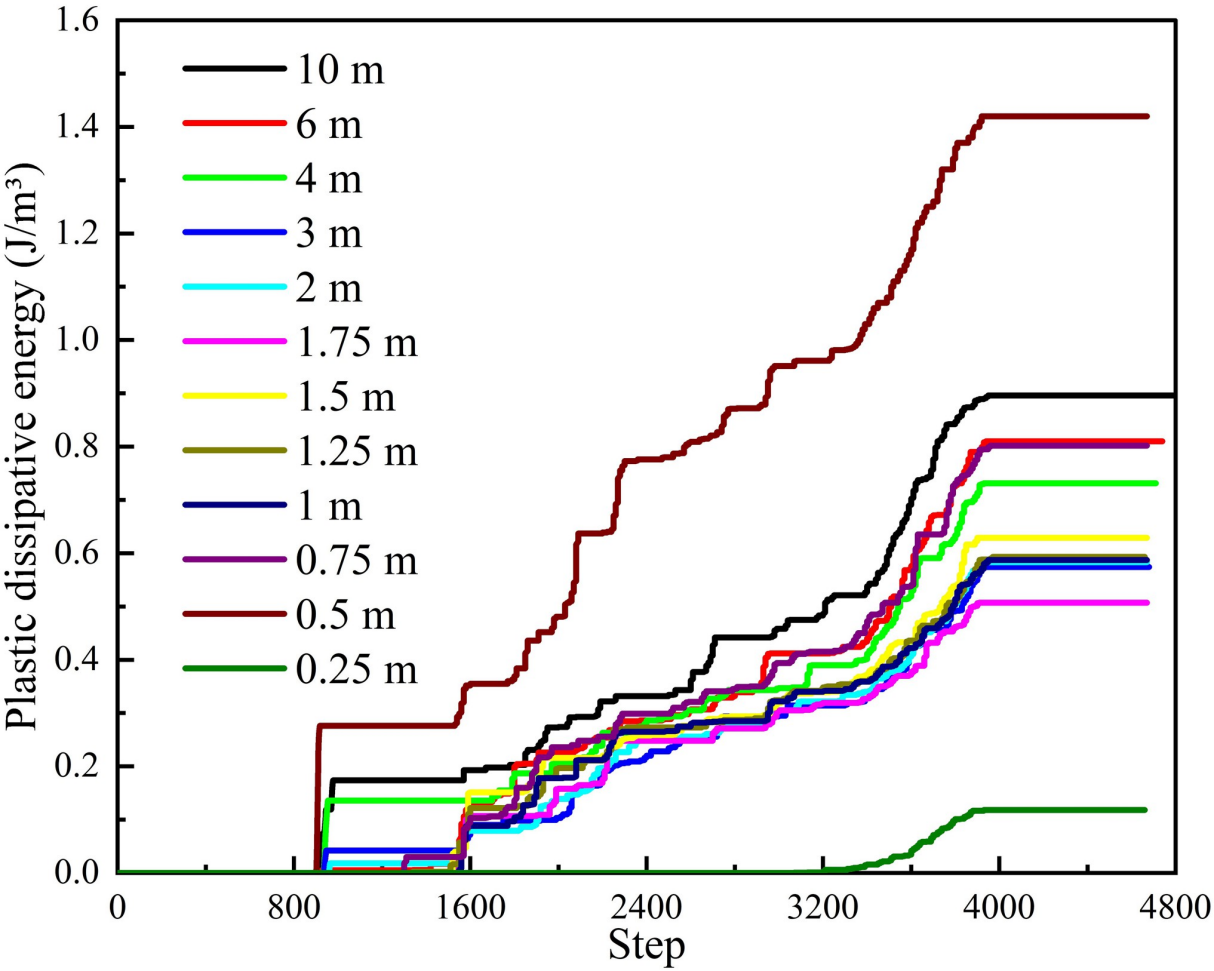
Energy dissipation law of different supporting position schemes.

**Fig 21:**
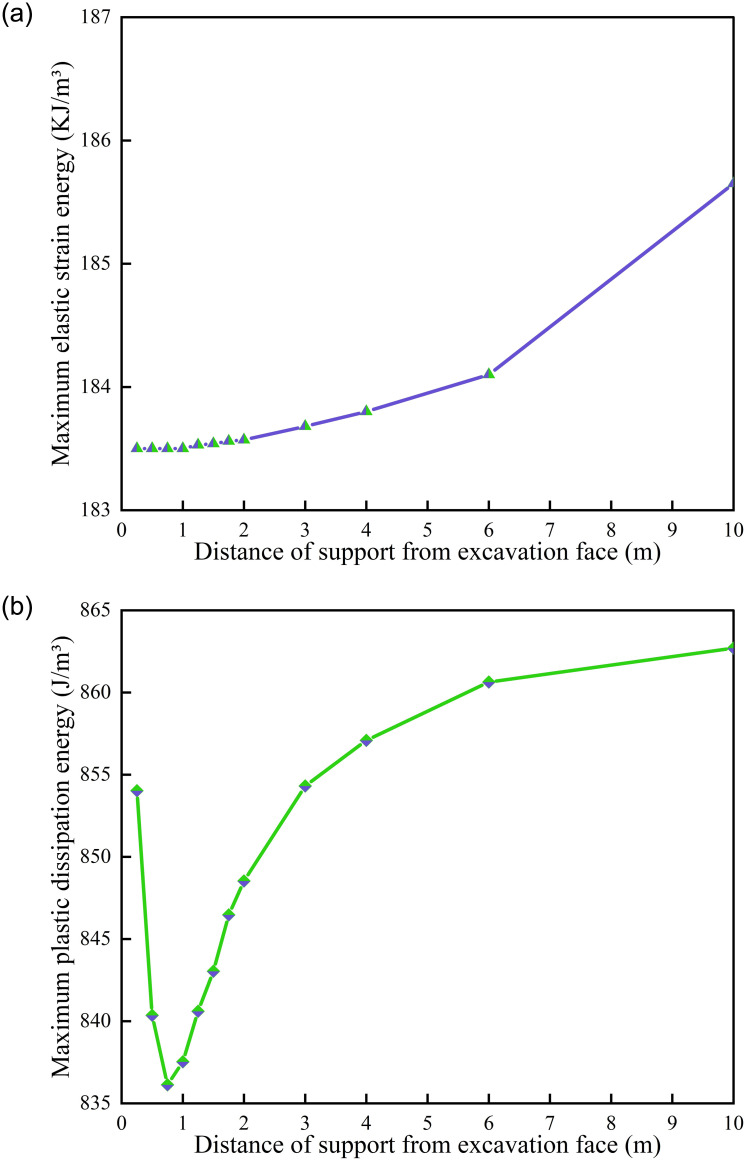
Energy dissipation of different supporting position schemes. (a) Maximum elastic strain energy, (b) Maximum plastic dissipated energy.

*(2) Support position*. Under different supporting positions, energy dissipation monitoring is carried out on the central position of the roadway excavation surface, as shown in Fig 20. According to Fig 20 that the dissipation energy in this area is noteworthy less than the value of the central position of the roadway initial surface. All schemes surge when the calculation reaches 900 steps, and the amplitude of different schemes is different. The schemes of 0.5 m, 10 m and 4 m have a large increase, while the other schemes are tiny. Except for 0.25 m scheme, other schemes generally follow the trend of "flatness-steep increase-flatness-growth-flatness". From the overall energy dissipation value, 0.5 m > 10 m > 6 m > 0.75 m > 4 m > 1.5 m > 1.25 m > 1 m > 2 m > 3 m > 1.75 m > 0.25 m. Among them, 0.5 m is significantly higher than other schemes, 0.25 m is significantly lower than other schemes. Meanwhile, other schemes are close to each other as a whole. The maximum energy dissipation of different supporting position schemes in the model is shown in Fig 21. According to Fig 21(a), as the distance between the support structure and the working face increases gradually, the maximum elastic strain energy in the model also increases gradually. The maximum value is 185.65 KJ for a 10 m scheme and the minimum value is 183.50 KJ for a 0.25 m scheme. According to Fig 21(b), as the distance between the support structure and the working face gradually increases, the maximum plastic energy in the model presents a trend of "first decreasing and then increasing", which decreases to the lowest value at 0.75 m (836.13 J) and increases to the maximum value at 10 m (862.71 J).

## 4 Discussion

**Fig 22:**
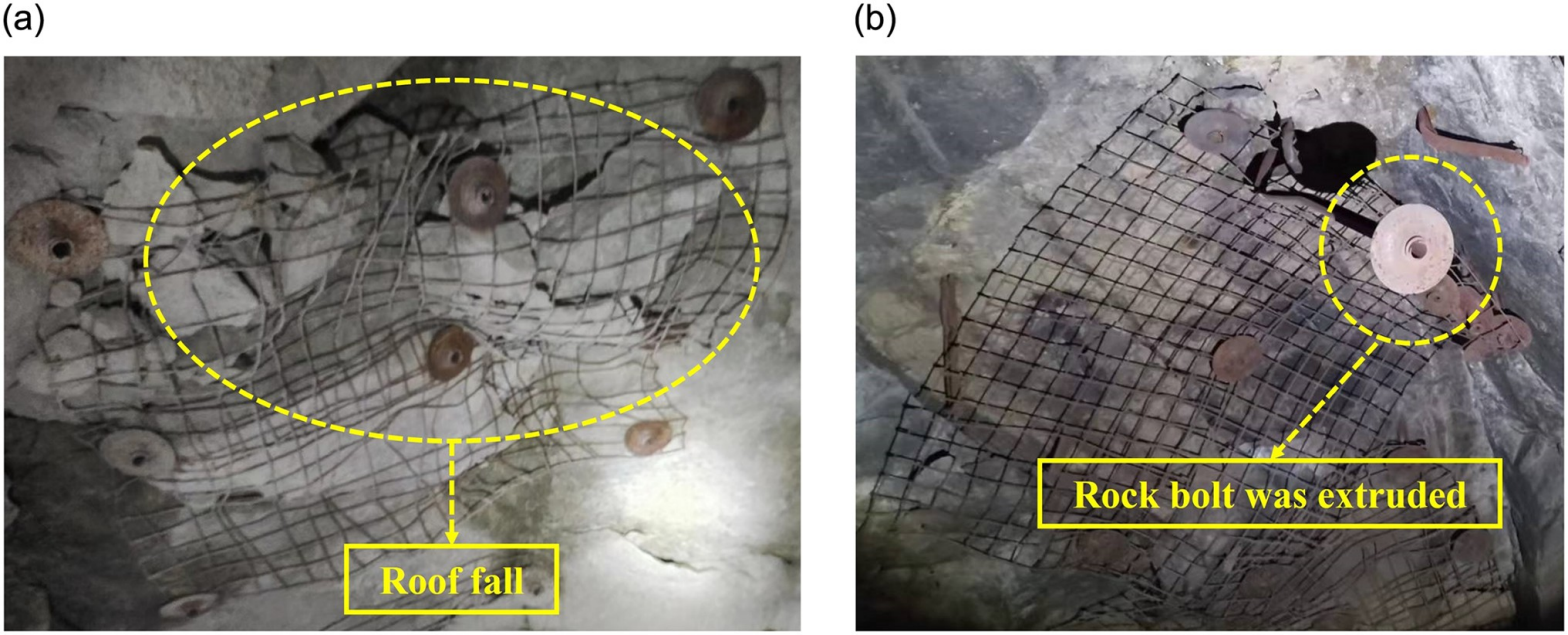
Failure condition of roadway support structure. (a) Roof fall, (b) rock bolt was extruded.

In the mining process, although the digging cost sometimes accounts for more than 50% of the total cost, it is still difficult to avoid the occurrence of underground engineering disasters [44]. Meanwhile, the "mining imbalance" is serious in mining, which restricts the improvement of the efficient production level of the mine. To solve these problems, some researchers focus on making new supporting structures, proposing more comprehensive supporting schemes, exploring more rapidly excavation technology, or developing more accurate safety warning systems. They reduce the possibility of secondary support and roadway damage to a certain extent. These research results improve the stability of roadway, but rarely touch the essence (energy) of roadway failure. Different from displacement, the energy in rock mass is difficult to be directly observed, but it is the conversion between these energy type that causes the negative phenomena such as roof caving, caving, rock-burst or support failure (Fig 22).

The time and space of supporting is "the most primitive" and "the most direct" factors affecting the stability of the roadway. At present, most of the relevant designs still use engineering analogy to determine their relevant values, which means that it only relies on relevant experience to carry out simple design. The Convergence-Confinement Method, on the other hand, starts from the macroscopic displacement changes and does not go deeper to analyze the essence of rock failure.

**Fig 23:**
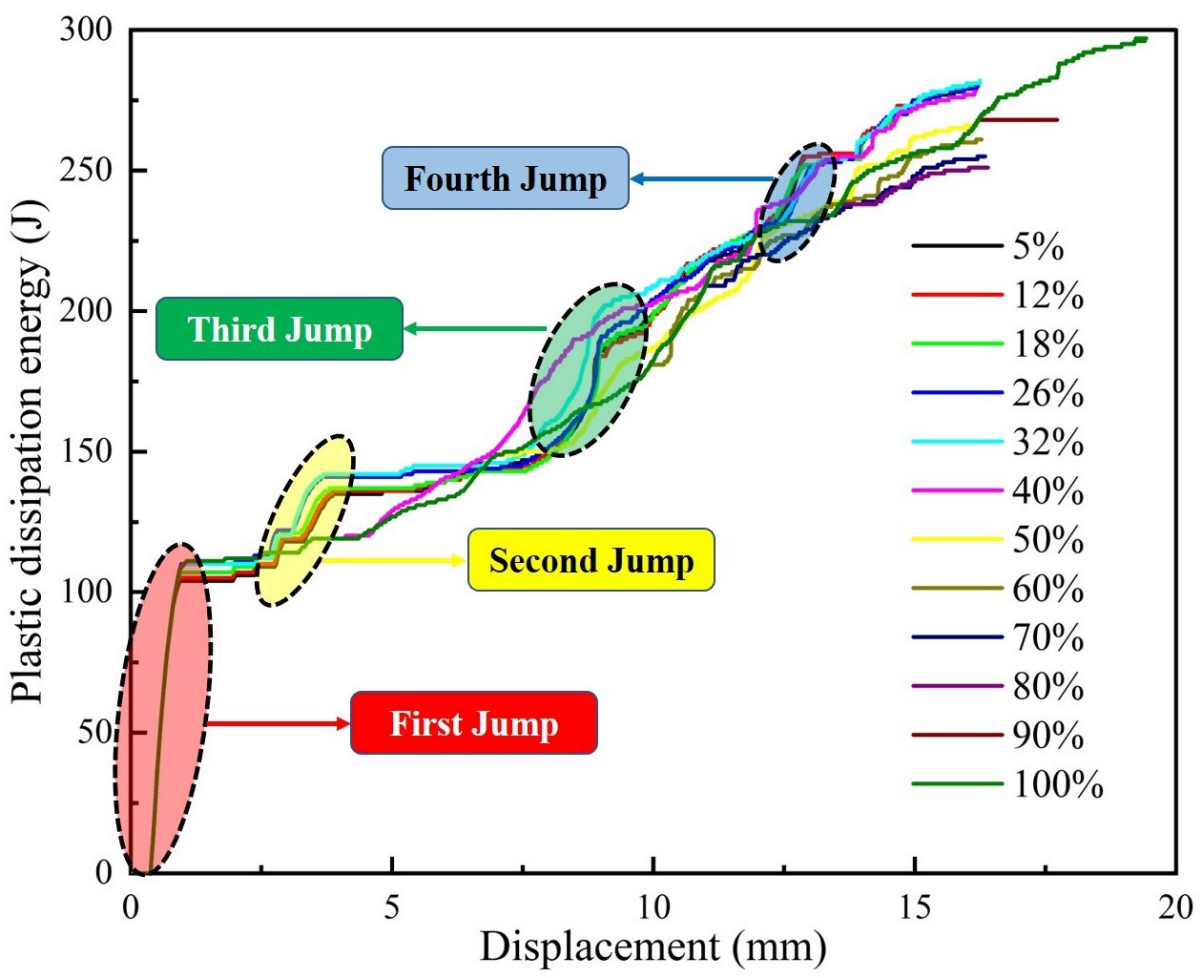
Displacement-dissipation energy curves of support schemes under different energy dissipation degrees.

In this study, based on the energy theory and numerical simulation software, twelve supporting schemes with different energy dissipation degrees are designed. We found through a series of simulations that energy dissipation has a "jump" and is not closely related to the change process of displacement. Displacement is the X axis and energy dissipation is the Y axis. Displacements and dissipation energy curves of supporting schemes with different energy dissipation degrees are obtained, as shown in Fig 23. According to Fig 23 that there are roughly four "jumps" in energy dissipation during the process of gradual increase of displacement, and about 110 J is generated at one time between the occurrence of maximum jump amplitude and the initial generation of displacement. Based on the calculation formula of plastic dissipation energy, it is preliminarily assumed that in surrounding rock, various stress and strain changes generated by different rock deformation values do not show linear correlation characteristics with the increase of displacement, which means that it is consistent with the typical stress-strain curve [45] before the peak of rock in mechanical tests, reflecting the elastic, plastic and viscous properties of rock.

Based on the above findings, a novel idea can be provided for the selection of roadway supporting time. It can be considered that supporting should be carried out before the second and second "jump" of roadway energy dissipation occur. Currently, a certain displacement is generated, reserving a certain time for roadway support, and the degree of plastic dissipation energy is tiny, and the accumulation of elastic strain energy is also tiny. Combined with Fig 1, this method can provide a basis for selecting μ_0_ in the Convergence-Confinement Method, solve the problem that it is difficult to determine the value of the initial reserved displacement of roadway (usually, the selection method of μ_0_ is the value of the displacement generated by the inner wall of roadway during supporting), and provide a reference for the selection of the initial supporting time in practical engineering.

**Fig 24:**
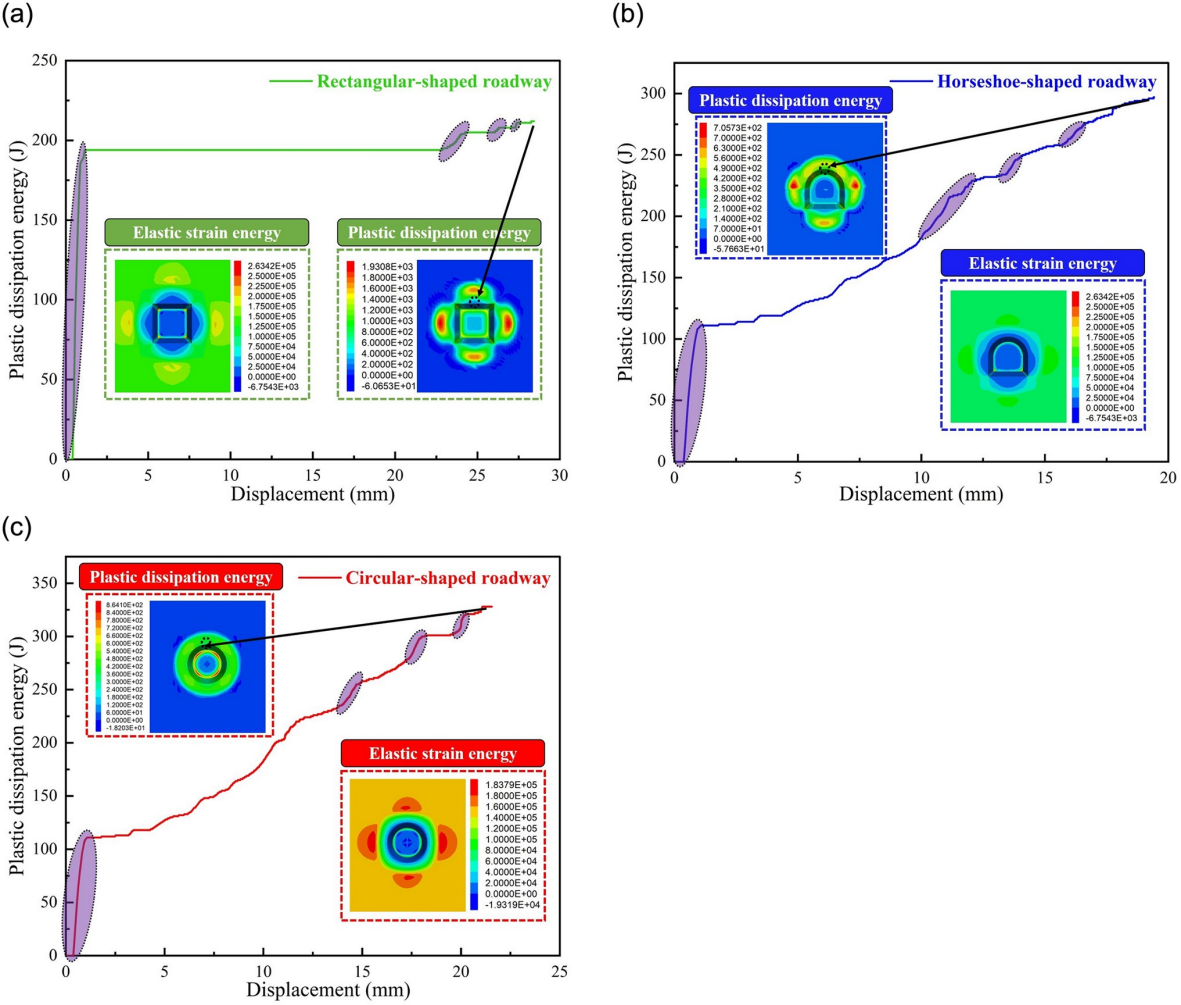
Displacement-dissipation energy curves of roadway with different sections (Unite: J). (a) rectangle, (b) Horseshoe shape, (c) circular.

Under the same physical and mechanical parameters and boundary conditions, additional rectangular and horseshoe roadway models were established, and the length and width of these two models were 3 m. Through simulation calculation, displace—dissipated energy curves of circular, rectangular and horseshoe roadway without support were obtained, as shown in Fig 24. It can be seen from the Fig 24. That rectangular roadway showed obvious "jump" characteristics. The accumulation degree of elastic strain energy is obviously greater than that of the circular and horseshoe shapes, and the plastic dissipated energy is the same. The order of elastic strain energy is rectangle > horseshoe > circle, and the accumulation degree of the circular strain energy is the lowest, and the possibility of impact accident is the least. The order of plastic dissipated energy is rectangle > horseshoe > circle, and the plastic dissipated energy is the lowest, and the surrounding rock properties of roadway are the least degraded. Likewise, the "jump" of horseshoe roadway without support is not significant except for the first jump, while the jump significance of circular roadway is less than that of rectangle but greater than that of horseshoe roadway.

Therefore, although there is an obvious "jump" phenomenon in some roadways, it is still worth discussing whether "jump" in plastic dissipated energy with the growth of displacement is an essential feature in different roadways. We need to further consider more factors such as roadway section type, physical and mechanical parameters of surrounding rock, ground stress, constitutive model, etc. Likewise, it is necessary to carry out similar simulation experiments to verify this feature.

Speculate on the above conclusions: in practical engineering applications, it is often impossible to carry out immediate support after roadway excavation. At this time, the generation of plastic zone and displacement is not complete, and there is a mass of reliable elastic strain energy stored in the roadway. Under the action of a certain incentive, a large amount of reliable strain energy can be converted into kinetic energy to cause roadway damage. Then it causes great pressure to the supporting structure, leading to the change of the supporting structure, and finally failure. When the support is too late, the energy dissipation degree is greater, although the displacement is released more, the surrounding rock parameters are seriously deteriorated, and the energy in the stress concentration area continues to accumulate. In this case, the stability of the roadway can be effectively controlled even if the support is carried out. Therefore, roadway support should not be carried out too late, otherwise, surrounding rock displacement is difficult to control even if the support is carried out. According to the results, the maximum displacement of roadway without support is 19.44 mm, and when the energy dissipation reaches 100%, the displacement of roadway also reaches 19.24 mm. The supporting effect (position control effect) is only 6% of that when energy dissipation reaches 40%, and the supporting effect is significantly reduced.

## 5 Conclusions

In this study, numerical simulation of roadway excavation support was carried out using FLAC3D to study the effect of different supporting time and supporting location on the stability of the roadway. The results show that:

1. The appropriate opportunity can effectively improve the effect of support with the same parameters, and the influence of energy dissipation (time) and support position (space) on the overall stability of the roadway is not a simple linear relationship, and the "moderate" stage of time and space achieves the great results.
2. The established "displacement-dissipation energy" shows that there is a significant dissipation energy "jump" phenomenon in the displacement growth of the circular roadway, as well as in other shapes of roadway excavation.
3. A method of determining the supporting opportunity of the roadway is proposed based on the "jump" phenomenon of dissipated energy, which provides a novel approach of idea for the design of roadway support programs.

## Supporting information

S1 File (ZIP)
